# The Interplay Between Strictness of Policies and Individuals’ Self-Regulatory Efforts: Associations with Handwashing During the COVID-19 Pandemic

**DOI:** 10.1093/abm/kaab102

**Published:** 2021-12-06

**Authors:** Aleksandra Luszczynska, Zofia Szczuka, Charles Abraham, Adriana Baban, Sydney Brooks, Sabrina Cipolletta, Ebrima Danso, Stephan U Dombrowski, Yiqun Gan, Tania Gaspar, Margarida Gaspar de Matos, Konstadina Griva, Michelle I Jongenelis, Jan Keller, Nina Knoll, Jinjin Ma, Mohammad Adbdul Awal Miah, Karen Morgan, William Peraud, Bruno Quintard, Vishna Shah, Konstantin Schenkel, Urte Scholz, Ralf Schwarzer, Maria Siwa, Diana Taut, Silvia C M Tomaino, Noa Vilchinsky, Hodaya Wolf

**Affiliations:** Wroclaw Faculty of Psychology, SWPS University of Social Sciences and Humanities, Wroclaw, Poland; Melbourne Centre for Behaviour Change, Melbourne School of Psychological Sciences, The University of Melbourne, Australia; Wroclaw Faculty of Psychology, SWPS University of Social Sciences and Humanities, Wroclaw, Poland; School of Psychology, Deakin University, Geelong, Australia; Department of Psychology, Babes-Bolyai University, Cluj-Napoca, Romania; Faculty of Kinesiology, University of New Brunswick, Fredericton, Canada; Department of General Psychology, University of Padova, Padova, Italy; Medical Research Council Unit—The Gambia at London School of Hygiene and Tropical Medicine, Serrekunda, Gambia; Faculty of Kinesiology, University of New Brunswick, Fredericton, Canada; School of Psychological and Cognitive Sciences, Peking University, Beijing, China; Institute of Environmental Health, Medical School, University of Lisbon, Lisbon, Portugal; Institute of Environmental Health, Medical School, University of Lisbon, Lisbon, Portugal; Lee Kong Chian School of Medicine, Nanyang Technological University, Singapore; Melbourne Centre for Behaviour Change, Melbourne School of Psychological Sciences, The University of Melbourne, Australia; Department of Education and Psychology, Freie Universität Berlin, Berlin, Germany; Department of Education and Psychology, Freie Universität Berlin, Berlin, Germany; School of Psychological and Cognitive Sciences, Peking University, Beijing, China; School of Medicine, Perdana University—Royal College of Surgeons in Ireland, Kuala Lumpur, Malaysia; School of Medicine, Perdana University—Royal College of Surgeons in Ireland, Kuala Lumpur, Malaysia; Department of Psychology, INSERM 1219, University of Bordeaux, Bordeaux, France; Department of Psychology, INSERM 1219, University of Bordeaux, Bordeaux, France; Department of Infectious Diseases, Environmental Health Group, London School of Hygiene and Tropical Medicine, London, UK; Department of Psychology, Applied Social and Health Psychology, University of Zurich , Zurich, Switzerland; Department of Psychology, Applied Social and Health Psychology, University of Zurich , Zurich, Switzerland; University Research Priority Program “Dynamics of Healthy Ageing”, University of Zurich , Zurich, Switzerland; Department of Education and Psychology, Freie Universität Berlin, Berlin, Germany; Wroclaw Faculty of Psychology, SWPS University of Social Sciences and Humanities, Wroclaw, Poland; Department of Psychology, Babes-Bolyai University, Cluj-Napoca, Romania; Department of General Psychology, University of Padova, Padova, Italy; Department of Psychology, Bar-Ilan University, Ramat-Gan, Israel; Department of Psychology, Bar-Ilan University, Ramat-Gan, Israel

**Keywords:** COVID-19, HAPA, Policies, Cross-country, Pandemic

## Abstract

**Background:**

Patterns of protective health behaviors, such as handwashing and sanitizing during the COVID-19 pandemic, may be predicted by macro-level variables, such as regulations specified by public health policies. Health behavior patterns may also be predicted by micro-level variables, such as self-regulatory cognitions specified by health behavior models, including the Health Action Process Approach (HAPA).

**Purpose:**

This study explored whether strictness of containment and health policies was related to handwashing adherence and whether such associations were mediated by HAPA-specified self-regulatory cognitions.

**Methods:**

The study (NCT04367337) was conducted among 1,256 adults from Australia, Canada, China, France, Gambia, Germany, Israel, Italy, Malaysia, Poland, Portugal, Romania, Singapore, and Switzerland. Self-report data on cross-situational handwashing adherence were collected using an online survey at two time points, 4 weeks apart. Values of the index of strictness of containment and health policies, obtained from the Oxford COVID-19 Government Response Tracker database, were retrieved twice for each country (1 week prior to individual data collection).

**Results:**

Across countries and time, levels of handwashing adherence and strictness of policies were high. Path analysis indicated that stricter containment and health policies were indirectly related to *lower* handwashing adherence via lower self-efficacy and self-monitoring. Less strict policies were indirectly related to higher handwashing adherence via higher self-efficacy and self-monitoring.

**Conclusions:**

When policies are less strict, exposure to the SARS-CoV-2 virus might be higher, triggering more self-regulation and, consequently, more handwashing adherence. Very strict policies may need to be accompanied by enhanced information dissemination or psychosocial interventions to ensure appropriate levels of self-regulation.

## Teaser

When COVID-19 public policies across 14 countries were less strict, people reported higher self-regulatory abilities and, subsequently, higher adherence to a protective behavior, handwashing.

## Introduction

Research on the reduction of SARS-CoV-2 transmission has indicated that early adoption of preventive behaviors such as use of face masks, physical distancing, contact tracing, case isolation, and handwashing is likely to be a cost-effective way of reducing COVID-19 morbidity [1]. Simple handwashing with soap and water or with an alcohol-based sanitizer is recommended by the World Health Organization (WHO) [2] because SARS-CoV-2 can survive for up to 9 hr on human skin [3]. Studies conducted at the beginning of the COVID-19 pandemic showed that handwashing adherence or sanitizing rates were as high as 97% in some populations and situations (e.g., older people, washing or sanitizing hands for 20 s after returning home [4]) and as low as 53% in others (e.g., young adults, washing or sanitizing hands after coughing or sneezing [5]).

The guidelines proposed by the WHO [2] specify ‘how’, ‘how long’, and ‘when’ to wash/sanitize hands. The recommendations stress the need to wash all surfaces of the hands (the ‘how’ rule) for 20 s (the ‘how long’ rule) and suggest washing/sanitizing hands in specific situations: before preparing food or eating and after using the toilet, blowing one’s nose, coughing, sneezing, touching garbage, or visiting public spaces (the ‘when’ rule) [2]. Previous studies have assessed handwashing in relation to ‘how’ and ‘how long’, but have only considered some preselected situations (e.g., after coughing, after returning home [4–6]), or focused on handwashing frequency [7] without reference to the situational context.

## Containment and Health Policies During the COVID-19 Pandemic

The declaration of the COVID-19 pandemic by the WHO in March 2020 was followed by the introduction of containment*-*related and health-related policies that aimed to contain the spread of the virus [8]. Containment policies included closing down public spaces (e.g., shopping malls and schools), cancelling public events, and restricting the gathering and movement of people (e.g., imposing curfews and restrictions on national and international travels). Health-related policies ranged from COVID-19 testing, contact tracing, and social distancing to information campaigns and promotion of (i) regular handwashing/sanitizing and (ii) the use of personal protective equipment such as face masks [8]. Stricter policies meant higher restrictions in movement, more COVID-19 testing, and more information or promotion campaigns targeting handwashing, masking, and social distancing. Thus, containment and health policies represent a mix of restriction policies (restricting individual choices) and promotion policies (guiding choices and providing information) [9].

During the COVID-19 pandemic, containment and health policies were varying across the countries in terms of their content and strictness [10]. Some governments enforced very strict containment and health policies early and maintained high levels of strictness over time to prevent future increases in COVID-19 cases (e.g., China), whereas other governments increased enforcement over time or in response to changes in case numbers (e.g., Italy) [10].

## The Health Action Process Approach as a Model Explaining Handwashing Adherence

The health action process approach (HAPA [11, 12]) is a model of motivational and volitional determinants of behavior change. As suggested by the HAPA, awareness of health-related risks is one of the prerequisites of motivation to engage in protective action [11, 12]. Outcome expectancies (i.e., positive social, self-evaluative, or health-related consequences of action) and self-efficacy (i.e., an individual’s belief in their capabilities to exercise control over action, including overcoming situational barriers [11, 12]) are also key foundations of individual motivation to achieve goals. Thus, risk perception, positive outcome expectancies, and self-efficacy collectively predict intention (an assessment of motivation strength). Forming a strong and stable intention is the transition between the motivational (wanting to act) and volitional (acting) stages. In the latter stage, other cognitions become important to action and action maintenance [11, 12]. Behavior is directly predicted by forming (i) action plans about when, where, and how to perform behavior and (ii) coping plans to overcome barriers that could derail intended actions [11, 12]. Volitional processes also include self-monitoring, which refers to awareness, recording, and evaluating performance of the intended action. Self-efficacy is also critical in sustaining action [11, 12].

The predictive validity of the HAPA has been confirmed in numerous studies, with the majority of research focusing on nutrition or physical activity [13]. A recent meta-analysis that used the HAPA to predict health behaviors confirmed the central role of self-efficacy, as well as the effects of outcome expectancies, intention, and planning (self-monitoring was not included in this meta-analysis; [14]). The HAPA model has also been shown to predict physical distancing in social interactions during the COVID-19 pandemic [15] as well as handwashing frequency prior to the COVID-19 pandemic [16], and during the beginning of the spread of SARS-CoV-2 in China (January-February 2020) [17]. Overall, the HAPA model offers good predictive validity of individual preventive actions.

## Relationships Between Policies, Self-regulation, and Protective Action Adherence

Social ecological models highlight the role of societal-level determinants of individual action [18, 19], for example, public health policies that may promote or hinder health behavior patterns [19]. It is likely that policy variation shapes individual action indirectly through changing individual beliefs. However, there is limited evidence showing how policies may operate together with self-regulatory cognitions to predict protective action [18, 19].

There are several ways in which the strictness of containment and health policies may impact individual action. First, strict containment and health policies may disrupt everyday routines and so render the focal action/s (e.g., handwashing) more salient, thereby reducing forgetting and strengthening adherence. Second, strict policies may be viewed as infringements of liberties [9] and, when perceived in this way, generate opposition and reduced motivation to follow the guidelines [20]. Third, less strict policies may increase the probability of being exposed to the virus (e.g., while travelling and during public events), thus prompting individuals to engage in more self-regulatory efforts to minimize the likelihood of SARS-CoV-2 infection. Finally, less strict policies may promote individual-level responsibility, bolstering motivation to protect oneself and one’s community [21].

## Study Aims

This longitudinal study investigated correlates of adherence to handwashing guidelines (issued by the WHO [2]) during the first wave of the COVID-19 pandemic (March–September 2020) in 14 countries within Europe, Asia, North America, Africa, and Australia. We investigated whether the level of strictness of containment and health policies would be indirectly related to handwashing adherence at a follow-up, with the self-regulatory HAPA variables operating as the mediators. In line with the HAPA model’s predictions [11, 12], the mediators included both motivational (risk perception, positive outcome expectancies, self-efficacy, and intention) and volitional phase variables (self-efficacy, planning, and self-monitoring).

## Methods

### Procedure

This observational study (see ClinicalTrials.Gov, #NCT04367337) was conducted in 14 countries: Australia, Canada, China, France, Gambia, Germany, Israel, Italy, Malaysia, Poland, Portugal, Romania, Singapore, and Switzerland. The countries were recruited until the following criteria were met: (i) representing different trajectories of the COVID-19 pandemic (e.g., low vs. high numbers of total cases during the data collection period, as reported by the WHO Coronavirus Disease Situation Reports [22]) and varying in the strictness of containment and health policies at T0 [10]; (ii) representing at least 5 continents; and (iii) at least one country with moderate to high values (.550–.800) of the Human Development Index (HDI) and at least one country with low HDI values (below .550), as defined by United Nations [23].

Data were collected between March and September 2020, after obtaining ethics clearance (following the institutional regulations in each study country) and preparing eight country/language versions of all study materials. Data were collected on (i) strictness of containment and health policies and (ii) individual-level cognitions and behavior. Data at both these levels were assessed at two time points, resulting in four data collection points across the study. First, Time 0 (T0) involved collection of data on strictness of policies. Second, Time 1 (T1) data collection of the HAPA-specified cognitions and handwashing adherence was conducted 1–7 days after T0. Third, Time 2 (T2) data collection on policies index values was conducted at 1 month after T1. Fourth, Time 3 (T3) repeat data collection of the HAPA-specified cognitions and handwashing adherence was conducted 1–7 days after T2.

Individual (sociodemographic, HAPA, and adherence) data were obtained (at T1 and T3) using a web-based questionnaire administered using the Qualtrics platform. The questionnaire took approximately 15 min to complete. At T1, snowball sampling was adopted as the main recruitment strategy, with social networks and university websites used to advertise the study. Links to the survey were posted online, together with information about the study aims and design. The only inclusion criterion was being ≥ 18 years old. Informed consent was obtained, and data were anonymized. There was no compensation for participation. Before starting the questionnaire, participants were provided with information regarding the WHO handwashing guidelines [2]. After obtaining T1 and T3 self-report data, T0 and T2 data on strictness of policies were collected retrospectively. Strictness of policies was assessed 1 week prior to each individual’s data collection date for their country.

Of the 6,397 potential respondents who provided consent, 333 (5.2%) reported sociodemographic data only and then withdrew from the study. After completing the T1 questionnaire, respondents were asked if they were willing to complete the survey again in a month’s time and, if so, to provide their email. A total of 2,399 provided an email address. An invitation to complete the T3 questionnaire was sent via email from the Qualtrics platform, followed by 2 weekly reminders.

## Participants

Data from 1,256 participants were available at T1 and T3. The largest subsamples were collected in Germany and Australia, and the smallest in Gambia and Singapore. The mean sample size per country was 89.7 (*SD* = 58.89, range from *n* = 13 to *n* = 210). Characteristics for the national subsamples are presented in Supplementary Table 1.

The longitudinal sample comprised 19.1% men and 80.2% women, aged 18–86 years (*M* = 37.13, *SD* = 15.67). The majority (77.9%) had a university education, 21.5% had completed a high school or a vocational education, and 0.6% had primary education. Self-assessed economic status indicated that 43.9% regarded their economic situation to be similar to the average in their country, 43.4% reported above-average economic status, and 12.7% reported below-average status. More than one-third of respondents (36.9%) were employed on a full-time basis, 19.5% were in part-time employment, 8.8% were retired, and 43.9% were unemployed or students. A share of 12.5% was employed as a health care specialist during the COVID-19 pandemic. Half (52.5%) of the participants were married or living with a romantic partner, 42.1% were single, and 5.4% were widowed or divorced/separated.

## Materials

### T1 and T3 Handwashing Adherence (the Outcome)

An 8-item measure assessing handwashing adherence was developed (based on previous tools [4, 5, 24]) to capture adherence across situations. The stem ‘During the previous week, I’ve usually washed my hands (for at least 20 seconds, all surfaces of the hands)’ was followed by the eight situational contexts specified in the WHO [2] guidelines: ‘Before, during, and after preparing food’, ‘Before eating food’, ‘Before and after caring for someone at home who is sick with vomiting or diarrhea’, ‘After using the toilet’, ‘After blowing my nose, coughing, or sneezing’, ‘After touching an animal, animal food, or animal waste,’ ‘After visiting public spaces’, and ‘When my hands were visibly dirty’. In case a respondent indicated that in the previous week they did not care for a sick person or did not touch an animal/animal food/animal waste, the respective item was removed from the mean item score value for this participant. Responses were provided on a scale ranging from 1 (strongly disagree) to 4 (strongly agree) (T1: *M* = 3.12, *SD* = 0.63, *α* =.84; T3: *M* = 3.34, *SD* = 0.54, *α* =.85).

### Strictness of COVID-19 Containment and Health Policies (T0 and T2)

Hale et al. [10] proposed a containment and health index developed for between-countries comparisons. The index is calculated as an additive of eight containment policies (e.g., restrictions on international travel, limits on gatherings, cancelling public events, and schools and universities closed) and six health policies (e.g., information campaigns on handwashing or social distancing, contact tracing after a diagnosis, and use facial covering outside the home). The 14 policies are coded to have equal values and combined into a total score of values ranging from 0 to 100, with higher levels representing stricter policies. The containment and health index is calculated for each country for each week since the beginning of the COVID-19 pandemic and available from the Oxford COVID-19 Government Response Tracker database [10]. Data were retrieved for the period of data collection (between March 25 and September 20, 2020). The index values of T0 and T2 were matched with the exact date when, and the country where, each individual’s data were collected at T1 and at T3, respectively. Mean weekly values were *M* = 73.93, *SD* = 10.87 at T0 and *M* = 67.44, *SD* = 9.55 at T2. The values of the index across the countries are presented in Supplementary Tables 2 and 3.

### HAPA Measures (T1 and T3)

Risk perception (T1) was assessed with two items (e.g., “Compared to an average person of your age and gender, what is your risk of coronavirus SARS-CoV-2 infection?”) obtained from Park et al. [25]. A 5-point response scale ranging from 1 (*very low*) to 5 (v*ery high*) was used (*M* = 2.70, *SD* = 0.77, *r* =.50, *p* <.001). For the remaining HAPA variables, a 4-point response was applied, ranging from 1 (*strongly disagree*) to 4 (*strongly agree*). Positive outcome expectancies (T1) were assessed with 3 items [16] (e.g., “If I wash my hands frequently every day in accordance with the WHO recommendations, then I would be proud of myself that I take care of my health”). The 4-item response scale ranged from 1 (*strongly disagree*) to 4 (*strongly agree*), *M* = 3.15, *SD* = 0.53, *α* =.66. Self-efficacy was assessed at T1 and T3 with 4 items [16] (e.g., “I am confident I can wash my hands in accordance with the WHO recommendations, even if it would be difficult to change my routines”), T1: *M* = 3.31, *SD* = 0.58, *α* =.85; T3: *M* = 3.16, *SD* = 0.62, *α* =.87. Intention (T1) was measured with 2 items [16] (e.g., “Today and for the next 2 weeks I intend to properly wash my hands [for at least 20 s, all surfaces of the hands] with soap and water or alcohol-based hand rub in various situations identified by the WHO [e.g., before, during and after preparing food])”, *M* = 3.03, *SD* = 0.70, *r* = .40, *p* < .01. Planning (T1 and T3) was assessed with three items [16] referring to action plans (e.g., “I have made a concrete and detailed plan for the next 24 h regarding how to wash my hands with soap and water or alcohol-based hand rub”) and three items addressing coping plans (e.g., “To keep my hands clean in various situations I made a concrete plan regarding what to do if soap and water are not available”), T1: *M* = 2.28, *SD* = 0.68, *α* = .83; T3: *M* = 2.30, *SD* = 0.67, *α* = .86. Self-monitoring (T1 and T3) was assessed with three items [26] (e.g., “I monitor regularly if I washed my hands before eating or after touching the garbage”), T1: *M* = 3.00, *SD* = 0.59, *α* =.60; T3: *M* = 3.01, *SD* = 0.56, *α* =.66.

### Individual- and Country-Level Control Variables (T1)

Individuals’ data referring to country of residence, age, gender, education, perceived economic status, and marital status were assessed at T1. Participants indicated their education with responses representing the following 4 levels: primary school, vocational education, completed high school, and higher education. Perceived economic status was measured with one item, “Compared to the average situation of a family in your country, what is the economic situation of your family?”, with responses ranging from 1 (*much above the average*) to 5 (*much below the average*). The Human Development Index (HDI) [23], capturing overall development, health, and education was obtained for the 14 countries.

## Data Analysis

The G*Power calculator simulating a multiple regression model was used to determine the sample size. As there is a lack of research on the effects of policies on the HAPA cognitions and protective behaviors, we assumed small effect sizes *f*^2^ = .02, power of .95, and Type I error rate of .05. The determined sample size was 1,300 participants. Analyses were performed using SPSS and IBM AMOS versions 26. Path analyses were conducted with maximum likelihood estimation. The indirect effects were evaluated with user-defined estimands function and reported as unstandardized effect coefficients and two-sided 95% bias-corrected confidence intervals (CI), calculated with 5,000 bootstraps.

Instead of relying on *p*-values alone, two-tailed 95% CI for direct and indirect effect coefficients were reported and considered when interpreting the findings [27]. In particular, two-tailed 95% CI were reported for direct effect coefficients in the path analysis and for the correlation analysis conducted in the total sample. The values of indirect effects coefficients do not allow for a valid quantitative indication of the effect value [28]. However, it has been suggested that their two-tailed 95% bias-corrected CI can be used to determine whether or not such indirect effects exist [28].

When evaluating model fit, a cut-off point of ≤.08 was applied for the root mean square error of approximation (RMSEA) and standardized root mean square residual (SRMR) and a cut-off point ≥.95 was used for the comparative fit index (CFI) and the normed fit index (NFI) [29]. Missing data were accounted for by using a full information maximum likelihood procedure (FIML [29]). Little’s MCAR test indicated that the missing data patterns were missing at random, Little’s *χ*^*2*^(19) = 23.72, *p* = .207. Mardia’s coefficient of multivariate normality indicated moderate non-normality (31.44).

**Fig. 1.**
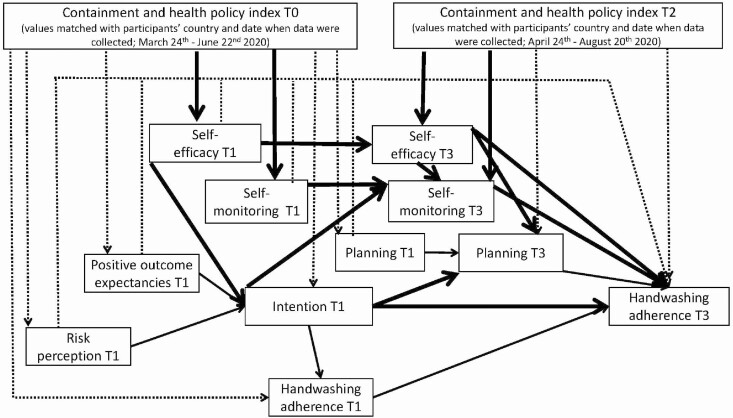
Results of path analysis for the mediation model: associations between the independent policies variables (T0, T2), the HAPA mediators (T1 and T3), and the dependent variable, handwashing adherence (T3). Dashed lines represent not-significant paths. Solid lines represent significant paths. Bold solid lines represent significant indirect effects.

The tested mediation model (see Fig. 1) represented a so-called half-longitudinal design [29]. The indirect effects of the half-longitudinal design [30] were obtained by controlling the effects of the T1 version of the putative mediator on the T3 putative mediator and the effects of the T1 version of the dependent variable on the T3 dependent variable. The model assumed that strictness of policies (T0) would be related to all T1 self-regulatory variables (risk perception, positive outcome expectancies, self-efficacy, intention, planning, and monitoring), handwashing adherence at T1, and strictness of policies at T2. T2 levels of strictness of policies were assumed to be related to the T3 self-regulatory variables (self-efficacy, planning, and self-monitoring) and adherence to handwashing at T3.

The pattern of associations among the self-regulatory HAPA variables (see Figure 1) is in line with Schwarzer’s theoretical proposal [11, 12]. The model included handwashing adherence at T1, which was assumed to be related to handwashing adherence at T3. Residuals of the HAPA variables at T1 and handwashing adherence at T1 were assumed to covary with each other (unless the two variables were linked with a regression path). The residuals of the HAPA variables at T3 were also assumed to covary.

Overall, 23 indirect effects linking strictness of policies (T0 or T2) with handwashing adherence (T3) were tested. There were seven single-mediator effects, linking the strictness of policies (T0; the independent variable) with the social cognitive variable (the mediator; T1 or T3 respectively), which in turn was associated with adherence to handwashing (the dependent variable; T3). There were 14 sequential mediating effects linking T0 strictness of policies with T3 handwashing adherence. For example, they included a sequence of mediators such as a motivational cognition at T1 (e.g., outcome expectancies) linked with intention as the second mediator (T1), which in turn could be related to the dependent variable, handwashing adherence (T3), or with the third mediator (planning or monitoring at T3). Finally, there were two indirect effects linking T2 strictness of policies (the independent variable) with adherence to handwashing (the dependent variable at T3) via self-efficacy (T3) as the first mediator and planning (T3) or monitoring (T3) as the second mediator.

Sensitivity analyses [31] were conducted to assess the robustness of the findings. First, analyses controlled for age, gender, and economic status. Second, analyses controlled for sociodemographic variables and mean number of COVID-19 cases in 14 days prior to data collection. Third, to test if the exclusion of dropouts affected the findings, the analyses were repeated with data obtained from both dropouts and completers.

Participants’ data are nested in *k* = 14 countries (with the sample sizes ranging across countries from 13 to 220 participants). Consequently, the intraclass correlation coefficient (ICC) was used to evaluate if there were significant clustering effects across the study variables. Next, we explored if the obtained associations occurring in one direction for the total sample (data from all 14 countries) may be significant and in the opposite direction when data from each country are analyzed separately. We explored patterns of associations specified in Figure 1 using correlation coefficients calculated for each country separately.

## Results

### Preliminary Analyses

We compared those who participated at T1 and agreed to take part in the follow-up questionnaire at the end of the T1 questionnaire, and provided an email address, but eventually did not respond to the follow-up questionnaire (*n* = 1,143) with those who responded at both T1 and T3 (*n* = 1,256). Those who completed T1 only and those who completed T1 and T3 assessments did not differ in T1 handwashing adherence, the T1 HAPA variables (risk perception, outcome expectancies, self-efficacy, intention, self-monitoring, and planning), and T0 index of strictness of policies (all *p*’s > .055). However, those who only completed T1 were younger, *F*(1, 2386) = 24.80, *p* < .001; *M* = 34.13, *SD* = 13.52 vs. *M* = 37.13, *SD* = 15.67, reported higher levels of economic status, *F*(1, 2386) = 24.80, *p* < .001; *M* = 2.73, *SD* = 0.82 vs. *M* = 2.66, *SD* = 0.83, and were more likely to be men, χ ^2^(1, *N* = 2,385) = 25.95, *p* < .001; 28.1% vs. 19.2%.

In the total sample (*N* = 1,256), adherence to handwashing was high at both measurement points (T1: *M* = 3.12, *SD* = 0.63; T3: *M* = 3.34, *SD* = 0.54) and did not change between T1 and T3, *F*(1, 1255) = 0.80, *p* = .372. The strictness of containment and health policies was high at T0 and T2, but there was a small reduction over time, *F*(1, 1255) = 202.90, *p* < .001; T0: *M* = 71.93, *SD* = 9.26 vs. T2: *M* = 67.44, *SD* = 9.55. Correlation coefficients (and their respective 95% CI) for the total sample are presented in Supplementary Table 4. Intraclass correlation coefficients, evaluating country-related clustering effects, were non-significant for the index of strictness of policies, HAPA variables, handwashing, and sociodemographic variables (see Supplementary Table 5).

## Indirect Associations Between Strictness of Policies, HAPA Variables, and Handwashing

The model had an acceptable fit, with *χ*^*2*^(27) = 189.139, *p* = .001, *χ*^*2*^/df = 7.01, NFI = .967, CFI = .971, RMSEA = .069 (90% CI: .060, .079), SRMR = .046. Direct associations between the independent policies variables (T0, T2), the HAPA mediators (T1, T3), and the dependent variable (T3) are presented in Fig. 1 and Table 1. The variables in the model explained 50.3% of handwashing adherence.

**Table 1.** Direct effects for the hypothesized model (*N =* 1,256)

Variables and hypothesized associations	Beta	95% lower CI for beta	95% upper CI for beta	p
Strictness of policies (T0)→ Risk Perception (T1)	−.004	−.054	.051	.898
Strictness of policies (T0)→ Outcome expectancies (T1)	−.005	−.064	.053	.858
Strictness of policies (T0)→ Self-efficacy (T1)	**−.068**	**−.120**	**−.012**	**.016**
Strictness of policies (T0)→ Intention (T1)	.005	−.040	.051	.846
Strictness of policies (T0)→ Planning (T1)	−.027	−.088	.029	.336
Strictness of policies (T0)→ Monitoring (T1)	**−.075**	**−.130**	**−.017**	**.008**
Strictness of policies (T0)→ Handwashing (T1)	−.024	−.076	.030	.331
Strictness of policies (T0) → Strictness of policies (T2)	**.295**	**.234**	**.354**	**<.001**
Risk Perception (T1)→ Intention (T1)	**.049**	**.004**	**.096**	**.037**
Risk Perception (T1)→ Handwashing (T3)	.019	−.023	.063	.344
Outcome expectancies (T1)→ Intention (T1)	**.270**	**.216**	**.323**	**<.001**
Outcome expectancies (T1)→ Handwashing (T3)	−.012	−.059	.039	.615
Self-efficacy (T1)→ Intention (T1)	**.394**	**.335**	**.449**	**<.001**
Self-efficacy (T1)→ Self-efficacy (T3)	**.526**	**.469**	**.576**	**<.001**
Self-efficacy (T1)→ Handwashing (T3)	.008	−.056	.067	.787
Intention (T1)→ Handwashing (T1)	**.716**	**.612**	**.815**	**<.001**
Intention (T1)→ Planning (T3)	**.076**	**.021**	**.130**	**<.001**
Intention (T1)→ Monitoring (T3)	**.132**	**.075**	**.191**	**<.001**
Intention (T1)→ Handwashing (T3)	**.059**	**.006**	**.113**	**.030**
Planning (T1)→ Planning (T3)	**.621**	**.576**	**.663**	**<.001**
Planning (T1)→ Handwashing (T3)	−.033	−.090	.025	.277
Monitoring (T1)→ Monitoring (T3)	**.465**	**.404**	**.521**	**<.001**
Monitoring (T1)→ Handwashing (T3)	−.042	−.101	.015	.143
Strictness of policies (T2)→ Self-efficacy (T3)	**−.095**	**−.140**	**−.050**	**<.001**
Strictness of policies (T2)→ Planning (T3)	−.024	−.065	.017	.228
Strictness of policies (T2)→ Monitoring (T3)	**−.049**	**−.090**	**−.007**	**.023**
Strictness of policies (T2)→ Handwashing (T3)	−.035	−.074	.004	.078
Self-efficacy (T3)→ Planning (T3)	**.152**	**.105**	**.199**	**<.001**
Self-efficacy (T3)→ Monitoring (T3)	**.226**	**.165**	**.286**	**<.001**
Self-efficacy (T3)→ Handwashing (T3)	**.157**	**.097**	**.221**	**<.001**
Planning (T3)→ Handwashing (T3)	**.057**	**.003**	**.114**	**.047**
Monitoring (T3)→ Handwashing (T3)	**.231**	**.173**	**.290**	**<.001**
Handwashing (T1)→ Handwashing (T3)	**.494**	**.429**	**.558**	**<.001**

*Note.* 95% CI = values of 95% two-tailed bias corrected confidence intervals. Indirect effect estimates presented in bold have values of two-tailed bias-corrected confidence intervals that do not include zero. T0 = Time 0; T1 = Time 1 (1–7 days later); T2 = Time 2 (one month after T1), T3= Time 3 (1–7 days after T2); Strictness of policies = Strictness of containment and health policies; Handwashing = Handwashing adherence index (based on the WHO guidelines). Data were collected in 14 countries (Australia, Canada, China, France, Gambia, Germany, Israel, Italy, Malaysia, Poland, Portugal, Romania, Singapore, and Switzerland) between March 25, 2020 and September 20, 2020, during the COVID-19 pandemic.

The observed pattern of associations among the self-regulatory HAPA variables (see Fig. 1, Table 1) corresponds to those assumed in the HAPA [11, 12] (see 95% CI in Table 1). The results indicated that higher levels of risk perception (T1), positive outcome expectancies (T1), and self-efficacy (T1) were related to a stronger intention (T1), which in turn was associated with higher levels of handwashing adherence assessed at T1 and at T3. Intention (T1) predicted higher levels of planning (T3) and self-monitoring (T3). In turn, higher levels of planning (T3) and self-monitoring (T3) were associated with higher levels of handwashing adherence (T3). Stronger self-efficacy (T3) was also associated with higher levels of self-monitoring (T3) and planning (T3). Consistent with the HAPA [11–13], T1 motivational variables were not directly related to handwashing adherence at T3. The values of covariance coefficients are presented in Supplementary Table 6.

Negative associations of small size were found between the strictness of containment and health policies (T0, T2) and the HAPA variables (see 95% CI; Table 1). Less strict policies (T0) were related to higher levels of T1 HAPA variables: self-efficacy (95% CI for β [−.12, −.01]) and self-monitoring (95% CI for β [−.13, −.02]). Similarly, less strict policies at T2 were related to higher levels of T3 self-efficacy (95% CI for β [−.14, −.05]) and T3 self-monitoring (95% CI for β [−.09, −.01]).

To investigate if the strictness of containment and health policies may be indirectly associated with handwashing, that is, mediated by the HAPA cognitions, 23 indirect effects analyses were conducted (see Table 2 for 95% CI indicating significance of the indirect effects). We found 10 significant indirect effects according to the 95% CIs. Strictness of policies at T0 explained adherence to handwashing (T3) via sequential mediators: (1) self-efficacy (T1) → intention (T1); (2) self-efficacy (T1) → intention (T1) → planning (T3); (3) self-efficacy (T1) → intention (T1) → self-monitoring (T3); (4) self-efficacy (T1) → self-efficacy (T3); (5) self-efficacy (T1) → self-efficacy (T3) → planning (T3); and (6) self-efficacy (T1) → self-efficacy (T3) → self-monitoring (T3). Furthermore, there were indirect effects of strictness of policies at T2 on handwashing (T3) via (7) self-efficacy (T3) and (8) self-monitoring (T3), operating as single mediators; but also via two sequential mediators: (9) self-efficacy (T3) → planning (T3) and (10) self-efficacy (T3) → self-monitoring (T3). Overall, less strict containment and health policies were related to higher levels of respective social cognitions. Thus, less strict policies (T0 and T2) were indirectly related to higher levels of handwashing adherence at T3. As the strictness of policies across the countries and time points was at least moderate, the ‘lower levels of strictness’ actually means at least moderate strictness. The results may be also read as showing the following pattern: higher levels of strictness of containment and health policies were related to lower levels of self-efficacy and self-monitoring (T1 and T3) which in turn was related to lower handwashing adherence (T3).

**Table 2.** Simple indirect effects in the hypothesized models (*N =* 1,256)

Simple indirect effects	Estimate^a^	*SE*	95%CI		
			**Lower**	**Upper**	***p***
Strictness of policies (T0)→ Risk perception(T1)→Handwashing(T3)	>−0.001	<0.001	>−0.001	<0.001	.699
Strictness of policies (T0)→Risk perception(T1)→Intention (T1)→Handwashing(T3)	>−0.001	<0.001	>−0.001	<0.001	.07
Strictness of policies (T0)→Risk perception(T1)→Intention (T1)→Planning(T3)→Handwashing(T3)	< 0.001	<0.001	>−0.001	<0.001	.635
Strictness of policies (T0)→Risk perception(T1)→Intention (T1)→Monitoring(T3)→Handwashing(T3)	>−0.001	<0.001	>−0.001	<0.001	.774
Strictness of policies (T0)→Outcome expectancies(T1)→Handwashing(T3)	<0.001	<0.001	>−0.001	<0.001	.687
Strictness of policies (T0)→Outcome expectancies(T1)→Intention (T1)→Handwashing(T3)	>−0.001	<0.001	>−0.001	<0.001	.731
Strictness of policies (T0)→Outcome expectancies(T1)→Intention (T1)→Planning (T3)→Handwashing(T3)	>−0.001	<0.001	>−0.001	<0.001	.656
Strictness of policies (T0)→Outcome expectancies(T1)→Intention (T1)→Monitoring(T3)→Handwashing(T3)	>−0.001	<0.001	>-0.001	<0.001	.819
Strictness of policies (T0)→Self-efficacy(T1)→Handwashing(T3)	>−0.001	<0.001	>−0.001	<0.001	.718
Strictness of policies (T0)→Self-efficacy(T1)→Intention (T1)→Handwashing(T3)	**>−0.001**	**<0.001**	**>−0.001**	**>−0.001**	**.019**
Strictness of policies (T0)→Self-efficacy(T1)→Intention (T1)→Planning (T3)→Handwashing(T3)	**>−0.001**	**<0.001**	**>−0.001**	**>−0.001**	**.015**
Strictness of policies (T0)→Self-efficacy(T1)→Intention (T1)→Monitoring (T3)→Handwashing(T3)	**>−0.001**	**<0.001**	**>−0.001**	**>−0.001**	**.007**
Strictness of policies (T0)→Self-efficacy (T1)→Self-efficacy(T3)→Handwashing(T3)	**>−0.001**	**<0.001**	**−0.001**	**>−0.001**	**.010**
Strictness of policies (T0)→Self-efficacy (T1)→Self-efficacy(T3)→Planning (T3)→Handwashing(T3)	**>−0.001**	**<0.001**	**>−0.001**	**>−0.001**	**.020**
Strictness of policies (T0)→Self-efficacy (T1)→Self-efficacy(T3)→Monitoring (T3)→Handwashing(T3)	**>−0.001**	**<0.001**	**>−0.001**	**>−0.001**	**.010**
Strictness of policies (T0)→Intention(T1)→Handwashing(T3)	<0.001	<0.001	>−0.001	<0.001	.729
Strictness of policies (T0)→Intention(T1)→Planning (T3)→Handwashing(T3)	<0.001	<0.001	>−0.001	<0.001	.662
Strictness of policies (T0)→Intention(T1)→Monitoring (T3)→Handwashing(T3)	<0.001	<0.001	>−0.001	<0.001	.833
Strictness of policies(T2)→Self-efficacy(T3)→Handwashing(T3)	−0.001	**<0.001**	**−0.001**	**>−0.001**	**<.001**
Strictness of policies(T2)→Self-efficacy(T3)→Planning (T3)→Handwashing(T3)	**>−0.001**	**<0.001**	**>−0.001**	**>−0.001**	**.024**
Strictness of policies(T2)→Self-efficacy(T3)→Monitoring (T3)→Handwashing(T3)	**>−0.001**	**<0.001**	**>−0.001**	**>−0.001**	**<.001**
Strictness of policies(T2)→Planning (T3)→ Handwashing(T3)	>−0.001	<0.001	>−0.001	<0.001	.140
Strictness of policies(T2)→Monitoring (T3)→ Handwashing(T3)	**−0.001**	**<0.001**	**>−0.001**	**>−0.001**	**.020**

*Note.* The values of the majority of indirect effect estimates were either larger than −0.001 (i.e., −0.0002) or smaller than 0.001 (i.e., 0.0002). Values of indirect effect estimates presented in bold have values of two-tailed-bias corrected 95% confidence intervals (CI) that do not include zero. T0 = Time 0; T1 = Time 1 (1–7 days later); T2= Time 2 (1 month after T1), T3 = Time 3 (1–7 days after T2); Strictness of policies = Strictness of containment and health policies; Handwashing = Handwashing adherence index (based on the WHO guidelines). Data were collected in 14 countries between March 25, 2020 and September 20, 2020, during the COVID-19 pandemic.

### Additional Findings: Sensitivity Analyses

Additional analyses included sociodemographic variables (age, gender, and economic status) as the predictors of handwashing adherence at T3 and correlates of all T1 variables. The model accounting for sociodemographic covariates had an acceptable fit, with *χ*^*2*^(45) = 265.524, *p* = .001, *χ*^*2*^/df = 5.70, NFI = .956, CFI = .963, RMSEA = .061 (90% CI: .045, .069), SRMR = .044. The pattern of findings was the same as in the models without these covariates (see Supplementary Tables 7–9). A second sensitivity analysis additionally accounted for the number of COVID-19 cases within 14 days prior to T1 and T3 (numbers matched with date and country of data collection; for measurement, see Supplementary Tables 10 and 11). The number of cases was assumed to relate to the strictness of policies (T0, T2), self-regulatory variables (T1, T3), and handwashing (T1, T3). A higher number of cases was related to stricter policies and higher self-monitoring (T1, T3) but unrelated to self-efficacy (T1, T3) and handwashing (T1, T3) (for 95% CI for standardized path coefficients see Supplementary Tables 10 and 11). Again, the patterns of associations between the independent, mediator, and dependent variables resembled those in the models without these covariates.

Third, it was tested if the missing data treatment strategy, namely, the deletion of non-completers, affected the patterns of observed associations (see Supplementary Tables 12–14). The path analysis was conducted in a sample of *N* = 2,339 participants, including both completers and dropouts. The model had an acceptable fit, with *χ*^*2*^(27) = 181.618, *p* = .001, *χ*^*2*^/df = 6.77, NFI = .987, CFI = .989, RMSEA = .049 (90% CI: .042, .056), SRMR =.029. Again, the same 10 indirect effects emerged, as found for the specified model calculated for completers only (*N* = 1,256). Three additional significant indirect effects were found, with strictness of policies (T0) being negatively associated with handwashing adherence (T3) via (1) intention (T1); (2) intention (T1) → planning (T3); and (3) intention (T1) → self-monitoring (T3).

In the next step, we explored if the bivariate associations that were significant in the mediation model and that represented the links between strictness of policies (T0, T2), HAPA variables (T1, T3), and handwashing at (T3) were significant and reverse when bivariate associations were calculated for each country separately. Compared to the findings obtained in the total sample, there were no cases of reverse effects regarding (i) the associations among HAPA variables and (ii) the associations between HAPA variables and T3 handwashing adherence (Supplementary Table 15). Regarding significant associations between strictness of policies (T0 and T2) and self-efficacy (T1) and planning (T3,), 2 out of 56 associations (3.6%) were reverse (i.e., significant and positive) in within-country correlations, compared to the findings for the model calculated in the total sample. Higher levels of strictness of policies (T2) were related to more frequent T3 planning in Germany (*r* = .18; *n* = 210; *p* = .010) and higher T3 self-efficacy in Singapore (*r* = .60, *n* = 13, *p* = .038).

## Discussion

Results provide evidence for indirect associations between strictness of public health containment and health policies introduced during the first wave of the COVID-19 pandemic and adherence to handwashing guidelines proposed by the WHO [2]. These associations were mediated by individual-level self-regulatory cognitions included in the HAPA model [11, 12].

The findings obtained for associations between the HAPA variables and handwashing are consistent with the theoretical assumptions of the HAPA [11, 12], as well as with the findings of a meta-analysis of research on the HAPA [14]. As suggested by the HAPA, risk perception and outcome expectancies were only indirectly linked with behavior. Self-efficacy played a significant role in both motivational and volitional phases. Additionally, we found that a facet of action control, namely, self-monitoring, was significantly associated with adherence to handwashing during the COVID-19 pandemic.

The findings, showing associations between strictness of containment and health policies and self-regulatory variables, provide support for social ecological models of health behavior patterns [18, 19]. Although the need for research testing models combining individual (e.g., self-regulation) and societal (e.g., policy) variables is often highlighted [19], to the best of our best knowledge, there is a lack of longitudinal research linking the societal variables (such as characteristics policies), individual mediators, and protective health behaviors. The direct associations between strictness of policies and the HAPA variables were small: for self-efficacy 95% CI for standardized path coefficient ranged from −.14 to −.01; for self-monitoring 95% CI for standardized path coefficient ranged from −.13 to −.01. Their clinical relevance is yet to be evaluated in research on public policy efficacy.

This study used an observational, correlational design, so causal inferences cannot be drawn. Instead, the findings provide a snapshot of a co-occurrence of strictness of complex containment and health policies, self-regulation processes, and handwashing during a specific period of the 6 months since the WHO has announced a pandemic and recommended handwashing as an important preventive strategy. This snapshot should be considered in the context of the generally high strictness of policies introduced across investigated countries and overall high average levels of handwashing adherence. The results may indicate that during the periods when containment and health policies were relatively less strict, respondents engaged in higher levels of self-monitoring/self-efficacy, which, in turn, were related to very high overall handwashing adherence. These results could mean that in a situation of less strict policies individuals may be motivated to invest more self-regulatory efforts to adhere to handwashing guidelines. When policies are less strict, exposure to the SARS-CoV-2 virus might be higher, triggering more self-regulation and consequently more handwashing adherence. Likewise, results may show that very high levels of strictness of governmental policies were related to relatively lower, yet moderate, levels of self-monitoring/self-efficacy, which translated to relatively lower, but still moderate-to-high adherence. These findings could mean that very strict policies may need to be accompanied by enhanced information dissemination or psychosocial interventions to ensure appropriate levels of self-regulation.

As indicated by reactance theory and research on reactance in the context of restrictive policies, the introduction of restrictive policies may result in increased deliberate processing of the situation and forming intentions to engage in “forbidden” behaviors [20]. Our findings suggest a different pathway, namely, an association between stricter COVID-19 policies and lower deliberate self-regulatory processes, which in turn were associated with lower handwashing adherence. It is possible that strictness of policies resulted in increased effortful self-regulation in relation to many everyday activities that during the pre- COVID-19 era were routine and/or habitually enacted (e.g., work, childcare, or social interactions). The increased cognitive load during the COVID-19 era, occurring due to the extensive self-regulation of daily activities such as social distancing [15], may have limited individuals’ capacity to self-regulate other behaviors, such as handwashing. This effect has been observed in experimental studies and is referred to as “ego-depletion” [32].

Alternatively, more lenient policies allowed more encounters of high-risk of infection situations during everyday life. This may have increased mastery of handwashing adherence and self-efficacy as well as prompted individuals to be more vigilant in monitoring, resulting in higher handwashing adherence. On the other hand, high strictness of policies might have resulted in such high restriction of population-level risk behaviors (with penalties imposed for breaking restrictions, in some cases [31]) that rendered people’s reliance on their own self-regulatory strategies obsolete. Pending replication of the negative correlation of policy strictness with self-regulation and protective behavior, future research should focus on these potential mediators to better understand what is driving these associations.

Another potential mediator explaining the negative indirect association between strictness of policies may be ‘pandemic fatigue’ [33], defined as a decline in motivation to engage in protective behaviors over time as the COVID-19 pandemic continues. Results obtained by Moore et al. [34] indicated that during the first 12 weeks of enforced COVID-19 containment policies, hand sanitizing increased initially, but for 10 following weeks the levels of sanitizing were low, even though the policies were still enforced. However, in terms of mean-level changes, the present study showed that although containment and health policies became less strict over time, handwashing adherence remained stable.

The findings indicated that all self-regulatory variables (e.g., risk perception, outcome expectancies, intention, and planning) were associated as predicted by the HAPA [11, 12]. For example, risk perception was associated with stronger intention. On the other hand, the indirect effects, linking strictness of policies, the HAPA cognitions, and handwashing adherence, were established for self-efficacy and self-monitoring only. Previous research conducted during the pandemic yielded similar findings, indicating that variables such as risk perception may be less consistently related to health behaviors [14, 15]. Our findings are also consistent with earlier research, indicating that across all HAPA cognitions, self-efficacy and self-monitoring are the most consistent predictors of health behavior during the pandemic, whereas other cognitions may be more likely to play a “low key” role [15].

The aforementioned explanations for the associations between policies and motivational or volitional variables are hypothetical and require an empirical investigation of the underlying processes. The present study captures the initial 6 months of the COVID-19 pandemic. It does not provide insights on relationships between policies and behavior patterns over longer time periods. Associations observed in the present study could change over time and as evidence for effectiveness of policies is observed by individuals.

Last but not least, the findings may result from the specificity of the applied policies index as well as the specificity of the protective behavior examined. The applied index combines multiple policies, including ‘lockdown’ restrictions and closures with measures such as testing policy, contact tracing, or promotion of protective behaviors [8]. Hence, high levels of the index of strictness of policies may result from very high levels of multiple containment policies combined with moderate or even low levels of promotion of or information on handwashing.

The present study has several limitations. Although the design was prospective, the HAPA variables and behavior were assessed at two time points which is a suboptimal approach in testing a mediation [30]. Due to the correlational nature, causal inferences are not possible. The sample included mostly women, and the majority of respondents had at least some university education and perceived their economic status as the average or above average in their respective country, which reduces the generalizability of findings. The dropout rates were high, and although there were no significant differences between the completers and dropouts in motivational social cognitions or behavior (T1), a large-scale dropout rate limits generalizations. Handwashing adherence was self-reported, but there is no recommended objective assessment of cross-situational handwashing and hand sanitizing (in contrast to an assessment conducted in one setting, which may be done measuring the use of hand sanitizer [34]). Data used in this study are clustered, with individuals nested in countries. This may have introduced non-independence in the sense that individuals living in the same country and being subject to similar policies at similar time points may be more similar than individuals across countries. However, because this study comprised too few clusters (i.e., countries, *N* = 14), a multi-level approach was not feasible. Given that the index of strictness of policies was country-specific and included in the analyses, this might have accounted partially for the violation of the non-independence requirement. Moreover, sample sizes differed across the included countries and cultural factors (such as personal beliefs of Muslims on the use of alcohol-based sanitizer) were not controlled.

Despite these limitations, the current study exhibits strengths including the use of a previously-validated, behavior-change theoretical model and assessment of public health policies when they were varying across the time span of the study and across the studied countries. Overall, this study provides a novel insight into complex interrelations between the strictness of containment-and-health policies, the HAPA-related social cognitions, and handwashing adherence during the COVID-19 pandemic. There is much more to learn about the mechanisms that may explain why higher strictness of policies is related to lower self-regulation. Replication of these results in other contexts is warranted.

## Supplementary Material

kaab102_suppl_Supplementary_Material
