# Supplementary material for: The Interplay Between Strictness of Policies and Individuals’ Self-Regulatory Efforts: Associations with Handwashing During the COVID-19 Pandemic
Source: Ann Behav Med. 2021 Dec 6;56(4):368–80. doi: 10.1093/abm/kaab102 (PMC8689736; doi:10.1093/abm/kaab102)
Supplement: kaab102_suppl_Supplementary_Material [file kaab102_suppl_supplementary_material.docx]

**The Interplay Between Strictness of Policies and Individuals’ Self-Regulatory Efforts: Associations with Handwashing During the COVID-19 Pandemic**

**Supplemental Materials**

Supplemental materials include:

- Information about the assessment of COVID-19 cases in 14-days prior to T1 and T3 measurement

- Supplementary Table 1, Sociodemographic Sample Characteristics at T1 for 14 Countries

- Supplementary Table 2, Strictness of Containment and Health Policies Across 14 Countries: Values at T1

- Supplementary Table 3, Strictness of Containment and Health Policies Across 14 Countries: Values at T2

- Supplementary Table 4, Correlation Coefficients for the Study Variables Included in the Model

- Supplementary Table 5, Estimates of Intraclass Correlation Coefficient for the Study Variables

- Supplementary Table 6, Covariances for the Mediation Model (*N* = 1,256)

- Supplementary Table 7, Sensitivity Analysis: Direct Effects for the Mediation Model (*N* = 1,256) Tested with Additional Covariates (Socioeconomic Status, Age, and Gender)

- Supplementary Table 8, Sensitivity Analysis: Indirect Effects for the Mediation Model (*N* = 1,256) Tested with Covariates (Socioeconomic Status, Age, and Gender)

- Supplementary Table 9, Sensitivity Analysis: Covariances for Mediation Model (*N* = 1,256) Tested with Additional Covariates (Socioeconomic Status, Age, and Gender)

- Supplementary Table 10, Sensitivity Analysis: Direct Effects for the Mediation Model with Basic Covariates (Socioeconomic Status, Age, and Gender) and an Additional Control Variable (Mean Number of COVID-19 Cases in 14 Days Prior to the Date of Data Collection)

- Supplementary Table 11, Sensitivity Analysis: Indirect effects for the Mediation Model with Basic Covariates (Socioeconomic Status, Age, and Gender) and an Additional Control Variable (Mean Number of COVID-19 Cases in 14 Days Prior to the Date of Data Collection)

- Supplementary Table 12, Sensitivity Analysis: Indirect Effects for the Mediation Model (*N* = 2,339) with Dropouts Included

- Supplementary Table 13, Sensitivity Analysis: Direct Effects for the Mediation Model (*N* = 2,339) with Dropouts Included

- Supplementary Table 14, Sensitivity Analysis: Covariances for the Mediation Model (*N* = 2,339) with Dropouts Included

- Supplementary Table 15, Correlation Coefficients for Associations Between the Variables Included in the Hypothetical Model, Calculated for Each Country Separately

**Assessment of COVID-19 Cases (Country-and-Day-Specific Data).** COVID-19 mortality and morbidity data for the 14 study countries for each day of data collection were extracted from the daily Coronavirus Disease Situation Reports prepared by the World Health Organization (2020). Reports from 20th January to 22nd July 2020 were used to identify: (i) the number of new COVID-19 cases per country per day recorded during the 14 days prior to data collection.

1. World Health Organization. Coronavirus disease (COVID-19) weekly epidemiological update and weekly operational update. Available at https://www.who.int/emergencies/diseases/novel-coronavirus-2019/situation-reports/. Accessed December 1, 2020.

**Supplementary Table 1**

*Sociodemographic Sample Characteristics at T1 for 14 Countries*

|  | AUS | CAN | CHIN | FRA | GAM | GER | ISR | ITA | MALA | POL | POR | ROM | SGP | SWI |
| --- | --- | --- | --- | --- | --- | --- | --- | --- | --- | --- | --- | --- | --- | --- |
|  |  |  |  |  |  |  |  |  |  |  |  |  |  |  |
| Number of participants | 195 | 66 | 127 | 107 | 23 | 210 | 89 | 63 | 36 | 65 | 56 | 78 | 13 | 128 |
| Mean Age (*SD*) | 45.54  (13.61) | 40.74 (18.36) | 23.14  (3.08) | 37.81  (14.60) | 36.04  (11.06) | 36.20  (17.10) | 48.42  (15.95) | 32.79  (15.93) | 33.89  (14.46) | 33.48  (11.96) | 38.68  (13.23) | 34.01  (12.16) | 42.92  (14.99) | 35.23  (15.13) |
| Gender (%) |  |  |  |  |  |  |  |  |  |  |  |  |  |  |
| Male | 5.6 | 24.2 | 26.8 | 14.0 | 65.2 | 21.4 | 20.2 | 25.4 | 19.4 | 15.4 | 25.0 | 15.4 | 15.4 | 19.5 |
| Female | 92.3 | 75.8 | 73.2 | 85.1 | 34.8 | 77.6 | 79.8 | 73.0 | 77.8 | 84.6 | 75.0 | 84.6 | 84.6 | 80.5 |
| Other | 2.1 | 0.00 | 0.00 | 0.9 | 0.0 | 1.0 | 0.0 | 1.6 | 2.8 | 0.0 | 0.0 | 0.0 | 0.0 | 0.0 |
| Education (%) |  |  |  |  |  |  |  |  |  |  |  |  |  |  |
| Primary education | 0.0 | 0.0 | 0.0 | 0.0 | 4.3 | 1.9 | 0.0 | 1.6 | 0.0 | 0.0 | 0.0 | 0.0 | 0.0 | 1.6 |
| High school | 12.3 | 6.1 | 4.7 | 15.0 | 4.4 | 7.6 | 5.6 | 17.5 | 5.6 | 23.1 | 5.4 | 10.3 | 15.4 | 10.2 |
| Vocational/post-secondary | 1.5 | 0.0 | 0.0 | 0.0 | 0.0 | 48.6 | 0.0 | 0.0 | 2.8 | 0.0 | 0.0 | 0.0 | 0.0 | 29.7 |
| University degree | 86.2 | 93.9 | 95.3 | 85.0 | 91.3 | 41.9 | 94.4 | 81.0 | 91.7 | 76.9 | 94.6 | 89.7 | 84.6 | 58.6 |
| Economic Status (%) |  |  |  |  |  |  |  |  |  |  |  |  |  |  |
| Below average | 14.4 | 4.5 | 25.2 | 19.6 | 21.7 | 7.1 | 29.2 | 14.3 | 8.3 | 6.2 | 1.8 | 2.6 | 7.7 | 7.8 |
| Average for family in a country | 39.4 | 45.5 | 53.5 | 40.2 | 56.6 | 44.3 | 18.0 | 57.1 | 44.5 | 32.3 | 53.6 | 55.1 | 38.5 | 47.7 |
| Above average | 46.2 | 50.0 | 21.3 | 40.2 | 21.7 | 48.6 | 52.8 | 28.6 | 47.2 | 61.5 | 44.6 | 42.3 | 53.8 | 44.5 |
| Marital status |  |  |  |  |  |  |  |  |  |  |  |  |  |  |
| Single/ Divorced/ Widowed | 26.7 | 59.1 | 85.0 | 45.8 | 30.4 | 47.8 | 23.6 | 68.3 | 63.9 | 27.5 | 37.5 | 37.2 | 46.2 | 60.2 |
| Living with a partner/ Civil Partnership/ Married | 73.3 | 40.9 | 15.0 | 54.2 | 69.6 | 52.2 | 76.4 | 31.7 | 36.1 | 72.5 | 62.5 | 62.8 | 53.8 | 39.8 |
| Employment |  |  |  |  |  |  |  |  |  |  |  |  |  |  |
| Unemployed/ student/ pensioner / retired | 31.3 | 37.9 | 80.3 | 34.6 | 13.0 | 56.7 | 27.0 | 69.4 | 58.3 | 32.1 | 19.6 | 32.1 | 23.1 | 39.1 |
| Full-time employment/ part-time employment | 68.7 | 62.1 | 19.7 | 65.4 | 87.0 | 43.3 | 73.0 | 30.6 | 41.7 | 67.9 | 80.4 | 67.9 | 76.9 | 60.9 |
| Healthcare professional |  |  |  |  |  |  |  |  |  |  |  |  |  |  |
| Health services professional | 9.7 | 16.3 | 6.2 | 24.4 | 36.4 | 6.7 | 5.6 | 9.5 | 35.0 | 6.2 | 21.6 | 11.9 | 18.2 | 18.7 |
| Other human services professional/ emergency worker/ Other | 90.3 | 83.7 | 93.8 | 75.6 | 63.6 | 93.3 | 94.4 | 90.5 | 65.0 | 93.8 | 78.4 | 88.1 | 81.8 | 81.3 |

*Note.* AUS = Australia; CAN = Canada; CHIN = China; FRA = France; GAM = Gambia; GER = Germany; ISR = Israel; ITA = Italy; MALA = Malaysia; POL = Poland; POR = Portugal; ROM = Romania; SGP = Singapore; SWI = Switzerland.

**Supplementary Table 2**

*Strictness of Containment and Health Policies Across 14 Countries: Values at T1*

| **Country** | ***N*** | **Mean** | **Standard Deviation** | **Minimum** | **Maximum** |
| --- | --- | --- | --- | --- | --- |
| Canada | 66 | 71.87 | 0.84 | 71.59 | 74.62 |
| China | 127 | 71.13 | 6.08 | 64.77 | 85.23 |
| France | 107 | 78.76 | 2.88 | 50.76 | 79.55 |
| Germany | 210 | 70.70 | 2.61 | 62.50 | 76.52 |
| Israel | 89 | 85.62 | 3.62 | 75.76 | 89.39 |
| Italy | 63 | 86.52 | 9.08 | 51.52 | 91.67 |
| Malaysia | 36 | 75.67 | 3.04 | 71.97 | 79.55 |
| Poland | 65 | 55.02 | 8.78 | 50.00 | 71.21 |
| Portugal | 56 | 73.89 | 2.46 | 71.97 | 85.61 |
| Singapore | 13 | 81.59 | 1.94 | 78.79 | 84.85 |
| Switzerland | 128 | 66.85 | 2.17 | 51.89 | 68.94 |
| Australia | 195 | 70.12 | 1.43 | 64.39 | 74.62 |
| Romania | 78 | 61.80 | 16.00 | 44.70 | 81.82 |
| Gambia | 23 | 72.75 | 2.73 | 70.08 | 75.76 |
| Total | 1256 | 71.93 | 9.27 | 44.70 | 91.67 |

**Supplementary Table 3**

*Strictness of Containment and Health Policies Across 14 Countries: Values at T2*

| **Country** | ***N*** | **Mean** | **Standard Deviation** | **Minimum** | **Maximum** |
| --- | --- | --- | --- | --- | --- |
| Canada | 66 | 69.54 | 1.32 | 68.56 | 73.11 |
| China | 127 | 77.08 | 9.97 | 64.77 | 85.23 |
| France | 107 | 69.71 | 8.80 | 50.76 | 79.55 |
| Germany | 210 | 66.06 | 4.61 | 62.50 | 76.52 |
| Israel | 89 | 75.17 | 2.07 | 68.94 | 78.03 |
| Italy | 63 | 71.48 | 11.97 | 51.52 | 91.67 |
| Malaysia | 36 | 70.73 | 7.63 | 59.85 | 79.55 |
| Poland | 65 | 68.46 | 6.42 | 47.73 | 71.21 |
| Portugal | 56 | 74.93 | 4.22 | 67.42 | 85.61 |
| Singapore | 13 | 63.64 | 9.26 | 57.58 | 81.82 |
| Switzerland | 128 | 66.21 | 4.68 | 48.48 | 73.11 |
| Australia | 195 | 61.30 | 5.70 | 51.89 | 82.95 |
| Romania | 78 | 50.79 | 7.91 | 42.26 | 71.97 |
| Gambia | 23 | 60.43 | 16.39 | 41.29 | 78.79 |
| Total | 1256 | 67.44 | 9.55 | 41.29 | 91.67 |

**Supplementary Table 4**

*Correlation Coefficients for the Study Variables Included in the Model*

| **Study variables** | **Correlation coefficients (Pearson’s *r*) and 95%CI** | | | | | | | | | | | | | |
| --- | --- | --- | --- | --- | --- | --- | --- | --- | --- | --- | --- | --- | --- | --- |
|  | 1 | 2 | 3 | 4 | 5 | 6 | 7 | 8 | 9 | 10 | 11 | 12 | 13 | 14 |
| 1. Strictness of Policies (T0) | 1 |  |  |  |  |  |  |  |  |  |  |  |  |  |
| 2. Risk Perception (T1) | -.004  [-.059, .053] | 1 |  |  |  |  |  |  |  |  |  |  |  |  |
| 3. Outcome expectancies (T1) | -.005  [-.062, .054] | -.026  [-.087, .038] | 1 |  |  |  |  |  |  |  |  |  |  |  |
| 4. Self-efficacy (T1) | -.068*  [-.122,  -.012] | .092**  [.034, .151] | .429**  [.373, .478] | 1 |  |  |  |  |  |  |  |  |  |  |
| 5. Intention (T1) | -.024  [-.079, .036] | .078**  [.021, .134] | .437**  [.387, .486] | .514**  [.460, .566] | 1 |  |  |  |  |  |  |  |  |  |
| 6. Planning (T1) | -.027  [-.085, .031] | .019  [-.039, .078] | .413**  [.361, .461] | .331**  [.277, .381] | .458**  [.410, .501] | 1 |  |  |  |  |  |  |  |  |
| 7. Self-monitoring (T1) | -.075**  [-.132,  -.017] | .104**  [.047, .161] | .358**  [.303, .412] | .441**  [.385, .497] | .506**  [.456, .554] | .446**  [.399, .492] | 1 |  |  |  |  |  |  |  |
| 8. Handwashing Adherence Index (T1) | -.041  [-.097, .016] | .112**  [.054, .166] | .301**  [.244, .356] | .416**  [.359, .472] | .519**  [.471, .563] | .329**  [.278, .377] | .494**  [.445, .542] | 1 |  |  |  |  |  |  |
| 9. Strictness of Policies (T2) | .295**  [.236, .354] | -.094**  [-.150,  -.037] | -.015  [-.072, .040] | -.091**  [-.149,  -.031] | -.023  [-.078, .032] | -.004  [-.060, .052] | -.068*  [-.123,  -.013] | -.041  [-.096, .015] | 1 |  |  |  |  |  |
| 10. Self-efficacy (T3) | -.090**  [-.144,  -.033] | .110**  [.054, .166] | .316**  [.257, .373] | .533**  [.478, .586] | .430**  [.375, .482] | .305**  [.253, .356] | .368**  [.309, .424] | .374**  [.314, .431] | -.142**  [-.196,  -.088] | 1 |  |  |  |  |
| 11. Planning (T3) | -.029  [-.090, .031] | .018  [-.038, .073] | .343**  [.287, .395] | .303**  [.248, .356] | .417**  [.365, .466] | .698**  [.666, .731] | .425**  [.376, .473] | .333**  [.283, .383] | -.049  [-.106, .005] | .370**  [.317, .421] | 1 |  |  |  |
| 12. Self-monitoring (T3) | -.072*  [-.129,  -.013] | .135**  [.080, .190] | .307**  [.253, .359] | .357**  [.303, .414] | .453**  [.406, .495] | .378**  [.327, .425] | .617**  [.573, .656] | .469**  [.425, .511] | -.113**  [-.168,  -.057] | .450**  [.391, .504] | .478**  [.433, .520] | 1 |  |  |
| 13. Handwashing Adherence Index (T3) | -.060*  [-.115,  -.003] | .124**  [.065, .181] | .271**  [.214, .326] | .387**  [.327, .444] | .464**  [.413, .511] | .304**  [.250, .335] | .435**  [.382, .485] | .663**  [.616, .704] | -.105**  [-.161,  -.049] | .462**  [.403, .516] | .366**  [.316, .415] | .543**  [.496, .587] | 1 |  |
| 14. Human Development Index  (HDI) | .022  [-.012, .056] | .173**  [.097, .247] | -.140**  [-.201,  -.077] | .052  [-.007, .117] | -.067*  [-.117,  -.012] | -.180**  [-.227,  -.131] | .032  [-.017, .082] | -.025  [-.084, .040] | -.095**  [-.180,  -.015] | .037  [-.018, .096] | -.173**  [-.219,  -.125] | .027  [-.018, .075] | -.018  [-.075, .044] | 1 |

*Note.* Two-tailed bias corrected 95% confidence intervals (CI) are reported in brackets. Strictness of Policies = Strictness of Containment and Health Policies.

** = Correlation is significant at the .01 level; * = Correlation is significant at the .05 level.

**Supplementary Table 5**

*Estimates of Intraclass Correlation Coefficient for the Study Variables*

| **Variable** | **All countries** | | | **Countries except for those with the lowest number of participants (n < 36; Gambia, Malaysia, Singapore)** | | |
| --- | --- | --- | --- | --- | --- | --- |
|  | **ICC value** | ***p*** | **95% CI (Lower, Upper)** | **ICC value** | ***p*** | **95% CI (Lower, Upper)** |
| Handwashing (T1) | .277 | .179 | -.467, .739 | .064 | .349 | -.346, .390 |
| Handwashing (T3) | -.485 | .775 | -2.013, .464 | -.006 | .490 | -.446, .344 |
| Strictness of Policies (T0) | .394 | .083 | -.229, .782 | .123 | .236 | -.261, .428 |
| Risk perception (T1) | -.841 | .883 | -2.735, .336 | -.471 | .962 | -1.116, .040 |
| Outcome expectancies (T1) | .220 | .234 | -.583, .719 | .201 | .113 | -.149, .479 |
| Self-efficacy (T1) | .265 | .191 | -.492, .725 | -.027 | .531 | -.477, .330 |
| Intention (T1) | .057 | .397 | -.913, .660 | .057 | .362 | -.355, .385 |
| Planning (T1) | -.008 | .459 | -1.045, .637 | -.132 | .710 | -.627, .262 |
| Self-monitoring (T1) | -.461 | .765 | -1.965, .473 | -.012 | .502 | -.455, .340 |
| Strictness of Policies (T3) | -.379 | .725 | -1.798, .503 | .333 | .014 | .041, .565 |
| Self-efficacy (T3) | -.047 | .494 | -1.125, .622 | -.017 | .512 | -.463, .337 |
| Planning (T3) | -2.658 | .993 | -6.422, -.319 | -.190 | .786 | -.710, .224 |
| Self-monitoring (T3) | -.672 | .841 | -2.392, .397 | -.135 | .715 | -.632, .260 |
| Age | -.034 | .483 | -1.098, .627 | -.010 | .499 | -.453, .341 |
| Gender | -.792 | .860 | -2.735, .389 | -.057 | .583 | -.534, .320 |
| Education | -.114 | .551 | -1.261, .598 | -.028 | .533 | -.479, .329 |
| Socioeconomic status | -1.554 | .965 | -4.181, .079 | -.108 | .673 | -.592, .278 |

*Note.* T0 = Time 0; T1 = Time 1 (1-7 days later); T2 = Time 2 (one month after T1); T3 = Time 3 (1-7 days after T2); ICC = Intraclass correlation coefficient; CI = Confidence intervals; Strictness of Policies = Strictness of Containment and Health Policies; Handwashing = Handwashing Adherence Index (based on the WHO Guidelines).

Data were collected in 14 countries (Australia, Canada, China, France, Gambia, Germany, Israel, Italy, Malaysia, Poland, Portugal, Romania, Singapore, and Switzerland) from 25.03.2020 to 20.09.2020.

**Supplementary Table 6**

*Covariances for the Mediation Model (N = 1,256)*

| **Covariances** | **Estimate** | ***SE*** | ***p*** |
| --- | --- | --- | --- |
| Outcome expectancies (T1) 🡨🡪 Risk perception (T1) | -0.011 | 0.012 | .353 |
| Self-efficacy (T1) 🡨🡪 Risk perception (T1) | 0.041 | 0.013 | .001 |
| Self-efficacy (T1) 🡨🡪 Outcome expectancies (T1) | 0.133 | 0.010 | < .001 |
| Planning (T1) 🡨🡪 Risk perception (T1) | 0.010 | 0.015 | .500 |
| Planning (T1) 🡨🡪 Outcome expectancies (T1) | 0.150 | 0.011 | < .001 |
| Planning (T1) 🡨🡪 Self-efficacy (T1) | 0.131 | 0.012 | < .001 |
| Monitoring (T1) 🡨🡪 Risk perception (T1) | 0.048 | 0.013 | < .001 |
| Monitoring (T1) 🡨🡪 Outcome expectancies (T1) | 0.112 | 0.009 | < .001 |
| Monitoring (T1) 🡨🡪 Self-efficacy (T1) | 0.150 | 0.011 | < .001 |
| Monitoring (T1) 🡨🡪 Planning (T1) | 0.178 | 0.012 | < .001 |
| Intention (T3)🡨🡪 Planning (T1) | 0.102 | 0.010 | < .001 |
| Handwashing (T1) 🡨🡪 Risk perception (T1) | 0.023 | 0.010 | .026 |
| Intention (T3)🡨🡪 Monitoring (T1) | 0.095 | 0.009 | < .001 |
| Planning (T3) 🡨🡪 Monitoring (T3) | 0.045 | 0.006 | < .001 |
| Handwashing (T1) 🡨🡪 Outcome expectancies (T1) | -0.004 | 0.007 | .631 |
| Handwashing (T1) 🡨🡪 Self-efficacy (T1) | 0.014 | 0.009 | .124 |
| Handwashing (T1) 🡨🡪 Intention (T3) | -0.080 | 0.014 | < .001 |
| Handwashing (T1) 🡨🡪 Monitoring (T1) | 0.041 | 0.009 | < .001 |

*Note.* T0 = Time 0; T1 = Time 1 (1-7 days later); T2= Time 2 (one month after T1); T3= Time 3 (1-7 days after T2); Handwashing = Handwashing Adherence Index (based on the WHO Guidelines). Data were collected in 14 countries (Australia, Canada, China, France, Gambia, Germany, Israel, Italy, Malaysia, Poland, Portugal, Romania, Singapore, and Switzerland) from 25.03.2020 to 20.09.2020.

**Supplementary Table 7a**

*Sensitivity Analysis: Direct Effects for the Mediation Model (N = 1,256) Tested with Additional Covariates (Socioeconomic Status, Age, and Gender)*

| **Variables and hypothesized associations** | **Beta** | **95% lower CI for beta** | **95% upper CI for beta** | ***p*** |
| --- | --- | --- | --- | --- |
| Strictness of Policies (T0) 🡪 Risk perception (T1) | -.003 | -.055 | .049 | .924 |
| Strictness of Policies (T0) 🡪 Outcome expectancies (T1) | -.005 | -.062 | .050 | .850 |
| Strictness of Policies (T0) 🡪 Self-efficacy (T1) | **-.067** | **-.120** | **-.013** | **.017** |
| Strictness of Policies (T0) 🡪 Intention (T1) | .005 | -.039 | .050 | .846 |
| Strictness of Policies (T0) 🡪 Planning (T1) | -.025 | -.083 | .033 | .372 |
| Strictness of Policies (T0) 🡪 Monitoring (T1) | **-.075** | **-.132** | **-.017** | **.008** |
| Strictness of Policies (T0) 🡪 Handwashing (T1) | -.025 | -.075 | .027 | .319 |
| Strictness of Policies (T0) 🡪 Strictness of Policies (T2) | **.295** | **.231** | **.354** | **< .001** |
| Risk perception (T1) 🡪 Intention (T1) | **.049** | **.004** | **.094** | **.037** |
| Risk perception (T1) 🡪 Handwashing (T3) | .018 | -.022 | .065 | .372 |
| Outcome expectancies (T1) 🡪 Intention (T1) | **.270** | **.214** | **.325** | **< .001** |
| Outcome expectancies (T1) 🡪 Handwashing (T3) | -.008 | -.060 | .037 | .728 |
| Self-efficacy (T1) 🡪 Intention (T1) | **.394** | **.336** | **.448** | **< .001** |
| Self-efficacy (T1) 🡪 Self-efficacy (T3) | **.526** | **.470** | **.576** | **< .001** |
| Self-efficacy (T1) 🡪 Handwashing (T3) | .005 | -.053 | .064 | .860 |
| Intention (T1) 🡪 Handwashing (T1) | **.717** | **.616** | **.820** | **< .001** |
| Intention (T1) 🡪 Planning (T3) | **.079** | **.025** | **.129** | **< .001** |
| Intention (T1) 🡪 Monitoring (T3) | **.135** | **.075** | **.191** | **< .001** |
| Intention (T1) 🡪 Handwashing (T3) | **.059** | **.008** | **.116** | **.030** |
| Planning (T1) 🡪 Planning (T3) | **.619** | **.577** | **.661** | **< .001** |
| Planning (T1) 🡪 Handwashing (T3) | -.035 | -.090 | .024 | .237 |
| Monitoring (T1) 🡪 Monitoring (T3) | **.465** | **.405** | **.522** | **< .001** |
| Monitoring (T1) 🡪 Handwashing (T3) | -.035 | -.097 | .020 | .214 |
| Handwashing (T1) 🡪 Handwashing (T3) | **.501** | **.428** | **.556** | **< .001** |
| Strictness of Policies (T2) 🡪 Self-efficacy (T3) | **-.095** | **-.142** | **-.050** | **< .001** |
| Strictness of Policies (T2) 🡪 Planning (T3) | -.023 | -.066 | .017 | .246 |
| Strictness of Policies (T2) 🡪 Monitoring (T3) | **-.049** | **-.091** | **-.008** | **.023** |
| Strictness of Policies (T2) 🡪 Handwashing (T3) | -.038 | -.075 | .005 | .059 |
| Self-efficacy (T3) 🡪 Planning (T3) | **.151** | **.106** | **.199** | **< .001** |
| Self-efficacy (T3) 🡪 Monitoring (T3) | **.224** | **.169** | **.287** | **< .001** |
| Self-efficacy (T3) 🡪 Handwashing (T3) | **.154** | **.098** | **.217** | **< .001** |
| Planning (T3) 🡪 Handwashing (T3) | **.058** | **.002** | **.113** | **.042** |
| Monitoring (T3) 🡪 Handwashing (T3) | **.227** | **.172** | **.290** | **< .001** |
| Age 🡪 Handwashing (T3) | -.004 | -.047 | .035 | .838 |
| Gender 🡪 Handwashing (T3) | -.011 | -.054 | .029 | .574 |
| Socioeconomic status 🡪 Handwashing (T3) | .010 | -.031 | .050 | .622 |

*Note.* Direct effect estimates presented in bold have values of two-tailed bias corrected confidence intervals that do not include zero. T0 = Time 0; T1 = Time 1 (1-7 days later); T2= Time 2 (one month after T1); T3= Time 3 (1-7 days after T2); Strictness of Policies = Strictness of Containment and Health Policies; Handwashing = Handwashing Adherence Index (based on the WHO Guidelines). Data were collected in 14 countries (Australia, Canada, China, France, Gambia, Germany, Israel, Italy, Malaysia, Poland, Portugal, Romania, Singapore, and Switzerland) from 25.03.2020 to 20.09.2020.

**Supplementary Table 8**

*Sensitivity Analysis: Indirect Effects for the Mediation Model (N = 1,256) Tested with Covariates (Socioeconomic Status, Age, and Gender)*

| **Simple indirect effects** | **Estimate** | ***SE*** | **95%CI** | |  |
| --- | --- | --- | --- | --- | --- |
|  |  |  | **Lower** | **Upper** | ***p*** |
| Strictness of Policies (T0)🡪Risk perception (T1)🡪Handwashing (T3) | >-0.001 | <0.001 | >-0.001 | <0.001 | .736 |
| Strictness of Policies (T0)🡪Risk perception (T1)🡪Intention (T1)🡪Handwashing (T3) | >-0.001 | <0.001 | >-0.001 | <0.001 | .756 |
| Strictness of Policies (T0)🡪Risk perception (T1)🡪Intention (T1)🡪Planning(T3)🡪Handwashing (T3) | 0.000 | <0.001 | >-0.001 | 0.000 | .719 |
| Strictness of Policies (T0)🡪Risk perception (T1)🡪Intention (T1)🡪Monitoring(T3)🡪Handwashing (T3) | >-0.001 | <0.001 | >-0.001 | 0.000 | .819 |
| Strictness of Policies (T0)🡪Outcome expectancies (T1)🡪Handwashing (T3) | >-0.001 | <0.001 | >-0.001 | <0.001 | .740 |
| Strictness of Policies (T0)🡪Outcome expectancies (T1)🡪Intention (T1)🡪Handwashing (T3) | >-0.001 | <0.001 | >-0.001 | <0.001 | .720 |
| Strictness of Policies (T0)🡪Outcome expectancies (T1)🡪Intention (T1)🡪Planning (T3)🡪Handwashing (T3) | >-0.001 | <0.001 | >-0.001 | <0.001 | .657 |
| Strictness of Policies (T0)🡪Outcome expectancies (T1)🡪Intention (T1)🡪Monitoring(T3)🡪Handwashing (T3) | >-0.001 | <.001 | >-0.001 | <0.001 | .810 |
| Strictness of Policies (T0)🡪Self-efficacy (T1)🡪Handwashing (T3) | >-0.001 | <0.001 | >-0.001 | <0.001 | .792 |
| Strictness of Policies (T0)🡪Self-efficacy (T1)🡪Intention (T1)🡪Handwashing (T3) | **>-0.001** | **<0.001** | **>-0.001** | **>-0.001** | **.019** |
| Strictness of Policies (T0)🡪Self-efficacy (T1)🡪Intention (T1)🡪Planning (T3)🡪Handwashing (T3) | **>-0.001** | **<0.001** | **>-0.001** | **>-0.001** | **.014** |
| Strictness of Policies (T0)🡪Self-efficacy (T1)🡪Intention (T1)🡪Monitoring (T3)🡪Handwashing (T3) | **>-0.001** | **<0.001** | **>-0.001** | **>-0.001** | **.007** |
| Strictness of Policies (T0)🡪Self-efficacy (T1)🡪Self-efficacy(T3)🡪Handwashing (T3) | **>-0.001** | **<0.001** | **-0.001** | **>-0.001** | **.010** |
| Strictness of Policies (T0)🡪Self-efficacy (T1)🡪Self-efficacy(T3)🡪Planning (T3)🡪Handwashing (T3) | **>-0.001** | **<0.001** | **>-0.001** | **>-0.001** | **.019** |
| Strictness of Policies (T0)🡪Self-efficacy (T1)🡪Self-efficacy(T3)🡪Monitoring (T3)🡪Handwashing (T3) | **>-0.001** | **<0.001** | **>-0.001** | **>-0.001** | **.010** |
| Strictness of Policies (T0)🡪Intention (T1)🡪Handwashing (T3) | >-0.001 | <0.001 | >-0.001 | <0.001 | .726 |
| Strictness of Policies (T0)🡪Intention (T1)🡪Planning (T3)🡪Handwashing (T3) | >-0.001 | <0.001 | >-0.001 | <0.001 | .671 |
| Strictness of Policies (T0)🡪Intention (T1)🡪Monitoring (T3)🡪Handwashing (T3) | >-0.001 | <0.001 | >-0.001 | <0.001 | .833 |
| Strictness of Policies (T2)🡪Self-efficacy (T3)🡪Handwashing (T3) | **-0.001** | **<0.001** | **-0.001** | **>-0.001** | **.000** |
| Strictness of Policies (T2)🡪Self-efficacy (T3)🡪Planning (T3)🡪Handwashing (T3) | **>-0.001** | **<0.001** | **>-0.001** | **>-0.001** | **.021** |
| Strictness of Policies (T2)🡪Self-efficacy (T3)🡪Monitoring (T3)🡪Handwashing (T3) | **>-0.001** | **<0.001** | **>-0.001** | **>-0.001** | **.000** |
| Strictness of Policies (T2)🡪Planning (T3)🡪 Handwashing (T3) | >-0.001 | <0.001 | >-0.001 | <0.001 | .148 |
| Strictness of Policies (T2)🡪Monitoring (T3)🡪 Handwashing (T3) | **-0.001** | **<0.001** | **-0.001** | **>-0.001** | **.018** |

*Note.* The values of the majority of indirect effect estimates were either larger than -0.001 (i.e., -0.0002) or smaller than 0.001 (i.e., 0.0002). Indirect effect estimates presented in bold have values of two-tailed bias corrected confidence intervals that do not include zero. Each bootstrap was based on 5,000 repetitions. 95%CI = two-tailed 95% bias-corrected confidence intervals. BCI that do not include zero indicate a significant indirect effect. T0 = Time 0; T1 = Time 1 (1-7 days later); T2= Time 2 (one month after T1); T3= Time 3 (1-7 days after T2); Strictness of Policies = Strictness of Containment and Health Policies; Handwashing = Handwashing Adherence Index (based on the WHO Guidelines).

Data were collected in 14 countries (Australia, Canada, China, France, Gambia, Germany, Israel, Italy, Malaysia, Poland, Portugal, Romania, Singapore, and Switzerland) from 25.03.2020 to 20.09.2020.

**Supplementary Table 9**

*Sensitivity Analysis: Covariances for Mediation Model (N = 1,256) Tested with Additional Covariates (Socioeconomic Status, Age, and Gender)*

| **Covariances** | **Estimate** | ***SE*** | ***p*** |
| --- | --- | --- | --- |
| Outcome expectancies (T1) 🡨🡪 Risk perception (T1) | -0.011 | 0.012 | .340 |
| Self-efficacy (T1) 🡨🡪 Risk perception (T1) | 0.041 | 0.013 | .001 |
| Self-efficacy (T1) 🡨🡪 Outcome expectancies (T1) | 0.133 | 0.010 | < .001 |
| Intention (T1) 🡨🡪 Planning (T1) | 0.100 | 0.010 | < .001 |
| Intention (T1) 🡨🡪 Monitoring (T1) | 0.095 | 0.009 | < .001 |
| Planning (T1) 🡨🡪 Risk perception (T1) | 0.010 | 0.015 | .513 |
| Planning (T1) 🡨🡪 Outcome expectancies (T1) | 0.149 | 0.011 | < .001 |
| Planning (T1) 🡨🡪 Self-efficacy (T1) | 0.130 | 0.012 | < .001 |
| Monitoring (T1) 🡨🡪 Risk perception (T1) | 0.047 | 0.013 | < .001 |
| Monitoring (T1) 🡨🡪 Outcome expectancies (T1) | 0.112 | 0.009 | < .001 |
| Monitoring (T1) 🡨🡪 Self-efficacy (T1) | 0.150 | 0.011 | < .001 |
| Monitoring (T1) 🡨🡪 Planning (T3) | 0.177 | 0.012 | < .001 |
| Handwashing (T1) 🡨🡪 Risk perception (T1) | 0.023 | 0.010 | .028 |
| Handwashing (T1) 🡨🡪 Outcome expectancies (T1) | -0.004 | 0.007 | .621 |
| Handwashing (T1) 🡨🡪 Self-efficacy (T1) | 0.014 | 0.009 | .133 |
| Handwashing (T1) 🡨🡪 Intention (T1) | -0.080 | 0.014 | < .001 |
| Handwashing (T1) 🡨🡪 Monitoring (T1) | 0.040 | 0.009 | < .001 |
| Handwashing (T1) 🡨🡪 Age | -0.023 | 0.193 | .905 |
| Handwashing (T1) 🡨🡪 Gender | -0.005 | 0.005 | .352 |
| Handwashing (T1) 🡨🡪 Socioeconomic status | -0.021 | 0.010 | .047 |
| Planning (T3) 🡨🡪 Monitoring (T3) | 0.045 | 0.006 | < .001 |
| Age 🡨🡪 Strictness of Policies (T0) | 9.842 | 4.083 | .016 |
| Age 🡨🡪 Risk perception (T1) | 1.037 | 0.343 | .003 |
| Age 🡨🡪 Outcome expectancies (T1) | 0.219 | 0.234 | .350 |
| Age 🡨🡪 Self-efficacy (T1) | 0.801 | 0.254 | .002 |
| Age 🡨🡪 Planning (T3) | 1.454 | 0.292 | < .001 |
| Age 🡨🡪 Monitoring (T1) | 0.502 | 0.249 | .044 |
| Gender 🡨🡪 Strictness of Policies (T0) | 0.076 | 0.102 | .458 |
| Gender 🡨🡪 Risk perception (T1) | -0.003 | 0.009 | .760 |
| Gender 🡨🡪 Outcome expectancies (T1) | -0.010 | 0.006 | .076 |
| Gender 🡨🡪 Self-efficacy (T1) | -0.031 | 0.006 | < .001 |
| Gender 🡨🡪 Planning (T3) | -0.010 | 0.007 | .164 |
| Gender 🡨🡪 Monitoring (T1) | -0.001 | 0.006 | .823 |
| Socioeconomic status 🡨🡪 Strictness of Policies (T0) | 0.765 | 0.218 | < .001 |
| Socioeconomic status 🡨🡪 Risk perception (T1) | -0.022 | 0.018 | .223 |
| Socioeconomic status 🡨🡪 Outcome expectancies (T1) | -0.026 | 0.012 | .039 |
| Socioeconomic status 🡨🡪 Self-efficacy (T1) | -0.037 | 0.013 | .005 |
| Socioeconomic status 🡨🡪 Planning (T3) | -0.004 | 0.015 | .809 |
| Socioeconomic status 🡨🡪 Monitoring (T1) | -0.027 | 0.013 | .040 |

*Note.* T0 = Time 0; T1 = Time 1 (1-7 days later); T2= Time 2 (one month after T1); T3= Time 3 (1-7 days after T2); Strictness of Policies = Strictness of Containment and Health Policies; Handwashing = Handwashing Adherence Index (based on the WHO Guidelines). Data were collected in 14 countries (Australia, Canada, China, France, Gambia, Germany, Israel, Italy, Malaysia, Poland, Portugal, Romania, Singapore, and Switzerland) from 25.03.2020 to 20.09.2020

**Supplementary Table 10**

*Sensitivity Analysis: Direct Effects for the Mediation Model with Basic Covariates (Socioeconomic Status, Age, and Gender) and an Additional Control Variable (Mean Number of COVID-19 Cases in 14 Days Prior to the Date of Data Collection)*

| **Variables and hypothesized associations** | **Beta** | **95% lower CI for beta** | **95% upper CI for beta** | ***p*** |
| --- | --- | --- | --- | --- |
| Strictness of Policies (T0) 🡪 Risk perception (T1) | -.022 | -.076 | .031 | .442 |
| Strictness of Policies (T0) 🡪 Outcome expectancies (T1) | .007 | -.051 | .066 | .795 |
| Strictness of Policies (T0) 🡪 Self-efficacy (T1) | **-.063** | **-.119** | **-.007** | **.025** |
| Strictness of Policies (T0) 🡪 Intention (T1) | .005 | -.039 | .050 | .846 |
| Strictness of Policies (T0) 🡪 Planning (T1) | .013 | -.046 | .073 | .647 |
| Strictness of Policies (T0) 🡪 Monitoring (T1) | **-.101** | **-.161** | **-.042** | **< .001** |
| Strictness of Policies (T0) 🡪 Handwashing (T1) | -.047 | -.099 | .008 | .058 |
| Strictness of Policies (T0) 🡪 Strictness of Policies (T2) | **.293** | **.227** | **.353** | **< .001** |
| Risk perception (T1) 🡪 Intention (T1) | **.049** | **.004** | **.094** | **.037** |
| Risk perception (T1) 🡪 Handwashing (T3) | .017 | -.025 | .061 | .404 |
| Outcome expectancies (T1) 🡪 Intention (T1) | **.270** | **.214** | **.325** | **< .001** |
| Outcome expectancies (T1) 🡪 Handwashing (T3) | -.009 | -.056 | .041 | .719 |
| Self-efficacy (T1) 🡪 Intention (T1) | **.394** | **.335** | **.447** | **< .001** |
| Self-efficacy (T1) 🡪 Self-efficacy (T3) | **.525** | **.469** | **.574** | **< .001** |
| Self-efficacy (T1) 🡪 Handwashing (T3) | .005 | -.054 | .062 | .860 |
| Intention (T1) 🡪 Handwashing (T1) | **.742** | **.644** | **.844** | **< .001** |
| Intention (T1) 🡪 Planning (T3) | **.079** | **.027** | **.131** | **< .001** |
| Intention (T1) 🡪 Monitoring (T3) | **.133** | **.076** | **.193** | **< .001** |
| Intention (T1) 🡪 Handwashing (T3) | **.059** | **.009** | **.117** | **.030** |
| Planning (T1) 🡪 Planning (T3) | **.619** | **.576** | **.662** | **< .001** |
| Planning (T1) 🡪 Handwashing (T3) | -.035 | -.094 | .020 | .250 |
| Monitoring (T1) 🡪 Monitoring (T3) | **.464** | **.404** | **.521** | **< .001** |
| Monitoring (T1) 🡪 Handwashing (T3) | -.036 | -.093 | .024 | .212 |
| Handwashing (T1) 🡪 Handwashing (T3) | **.501** | **.436** | **.564** | **< .001** |
| Strictness of Policies (T2) 🡪 Self-efficacy (T3) | **-.096** | **-.144** | **-.052** | **< .001** |
| Strictness of Policies (T2) 🡪 Planning (T3) | -.023 | -.066 | .018 | .249 |
| Strictness of Policies (T2) 🡪 Monitoring (T3) | **-.053** | **-.095** | **-.010** | **.015** |
| Strictness of Policies (T2) 🡪 Handwashing (T3) | -.039 | -.079 | .001 | .052 |
| Self-efficacy (T3) 🡪 Planning (T3) | **.151** | **.105** | **.198** | **< .001** |
| Self-efficacy (T3) 🡪 Monitoring (T3) | **.224** | **.167** | **.285** | **< .001** |
| Self-efficacy (T3) 🡪 Handwashing (T3) | **.154** | **.095** | **.213** | **< .001** |
| Planning (T3) 🡪 Handwashing (T3) | **.059** | **.006** | **.114** | **.040** |
| Monitoring (T3) 🡪 Handwashing (T3) | **.226** | **.166** | **.281** | **< .001** |
| 2-week COVID-19 cases (T0) 🡪 Risk perception (T1) | **.069** | **.015** | **.123** | **.014** |
| 2-week COVID-19 cases (T0) 🡪 Outcome expectancies (T1) | -.046 | -.105 | .013 | .100 |
| 2-week COVID-19 cases (T0) 🡪 Self-efficacy (T1) | -.016 | -.070 | .038 | .573 |
| 2-week COVID-19 cases (T0) 🡪 Planning (T1) | **-.138** | **.043** | **.150** | **< .001** |
| 2-week COVID-19 cases (T0) 🡪 Monitoring (T1) | **.097** | **-.189** | **-.087** | **< .001** |
| 2-week COVID-19 cases (T0) 🡪 Handwashing (T1) | **.083** | **.038** | **.126** | **< .001** |
| 2-week COVID-19 cases (T0) 🡪 Handwashing (T3) | .001 | -.062 | .054 | .945 |
| 2-week COVID-19 cases (T2) 🡪 Strictness of Policies (T2) | **.056** | **.007** | **.106** | **.037** |
| 2-week COVID-19 cases (T2) 🡪 Self-efficacy (T3) | .023 | -.030 | .052 | .338 |
| 2-week COVID-19 cases (T2) 🡪 Planning (T3) | -.001 | -.044 | .025 | .964 |
| 2-week COVID-19 cases (T2) 🡪 Monitoring (T3) | **.046** | **.016** | **.079** | **.034** |
| 2-week COVID-19 cases (T2) 🡪 Handwashing (T3) | .011 | -.017 | .085 | .567 |
| Age 🡪 Handwashing (T3) | -.004 | -.044 | .037 | .837 |
| Gender 🡪 Handwashing (T3) | -.011 | -.053 | .030 | .568 |
| Socioeconomic status🡪 Handwashing (T3) | .010 | -.031 | .049 | .629 |

*Note.* Direct effect estimates presented in bold have values of two-tailed bias corrected confidence intervals that do not include zero. T0 = Time 0; T1 = Time 1 (1-7 days later); T2= Time 2 (one month after T1); T3= Time 3 (1-7 days after T2); Strictness of Policies = Strictness of Containment and Health Policies; Handwashing = Handwashing Adherence Index (based on the WHO Guidelines); Data were collected in 14 countries (Australia, Canada, China, France, Gambia, Germany, Israel, Italy, Malaysia, Poland, Portugal, Romania, Singapore, and Switzerland) from 25.03.2020 to 20.09.2020

**Supplementary Table 11**

*Sensitivity Analysis: Indirect effects for the Mediation Model with Basic Covariates (Socioeconomic Status, Age, and Gender) and an Additional Control Variable (Mean Number of COVID-19 Cases in 14 Days Prior to the Date of Data Collection)*

| **Simple indirect effects** | **Estimate** | ***SE*** | **95%BCI** | |  |
| --- | --- | --- | --- | --- | --- |
|  |  |  | **Lower** | **Upper** | ***p*** |
| Strictness of Policies (T0)🡪Risk perception (T1)🡪Handwashing (T3) | >-0.001 | <0.001 | >-0.001 | <0.001 | .310 |
| Strictness of Policies (T0)🡪Risk perception (T1)🡪Intention (T1)🡪Handwashing (T3) | 0.000 | <0.001 | >-0.001 | 0.000 | .196 |
| Strictness of Policies (T0)🡪Risk perception (T1)🡪Intention (T1)🡪Planning(T3)🡪Handwashing (T3) | 0.000 | <0.001 | 0.000 | 0.000 | .146 |
| Strictness of Policies (T0)🡪Risk perception (T1)🡪Intention (T1)🡪Monitoring(T3)🡪Handwashing (T3) | 0.000 | <0.001 | >-0.001 | 0.000 | .240 |
| Strictness of Policies (T0)🡪Outcome expectancies (T1)🡪Handwashing (T3) | 0.000 | <0.001 | >-0.001 | <0.001 | .699 |
| Strictness of Policies (T0)🡪Outcome expectancies (T1)🡪Intention (T1)🡪Handwashing (T3) | <0.001 | <0.001 | >-0.001 | <0.001 | .667 |
| Strictness of Policies (T0)🡪Outcome expectancies (T1)🡪Intention (T1)🡪Planning (T3)🡪Handwashing (T3) | 0.000 | <0.001 | 0.000 | <0.001 | .628 |
| Strictness of Policies (T0)🡪Outcome expectancies (T1)🡪Intention (T1)🡪Monitoring(T3)🡪Handwashing (T3) | 0.000 | <0.001 | >-0.001 | <0.001 | .780 |
| Strictness of Policies (T0)🡪Self-efficacy (T1)🡪Handwashing (T3) | >-0.001 | <0.001 | >-0.001 | <0.001 | .767 |
| Strictness of Policies (T0)🡪Self-efficacy (T1)🡪Intention (T1)🡪Handwashing (T3) | **>-0.001** | **<0.001** | **>-0.001** | **>-0.001** | **.023** |
| Strictness of Policies (T0)🡪Self-efficacy (T1)🡪Intention (T1)🡪Planning (T3)🡪Handwashing (T3) | **>-0.001** | **<0.001** | **>-0.001** | **0.000** | **.017** |
| Strictness of Policies (T0)🡪Self-efficacy (T1)🡪Intention (T1)🡪Monitoring (T3)🡪Handwashing (T3) | **>-0.001** | **<0.001** | **>-0.001** | **>-0.001** | **.014** |
| Strictness of Policies (T0)🡪Self-efficacy (T1)🡪Self-efficacy(T3)🡪Handwashing (T3) | **>-0.001** | **<0.001** | **-0.001** | **>-0.001** | **.019** |
| Strictness of Policies (T0)🡪Self-efficacy (T1)🡪Self-efficacy(T3)🡪Planning (T3)🡪Handwashing (T3) | **>-0.001** | **<0.001** | **>-0.001** | **0.000** | **.022** |
| Strictness of Policies (T0)🡪Self-efficacy (T1)🡪Self-efficacy(T3)🡪Monitoring (T3)🡪Handwashing (T3) | **>-0.001** | **<0.001** | **>-0.001** | **>-0.001** | **.021** |
| Strictness of Policies (T0)🡪Intention (T1)🡪Handwashing (T3) | >-0.001 | <0.001 | >-.0001 | <0.001 | .714 |
| Strictness of Policies (T0)🡪Intention (T1)🡪Planning (T3)🡪Handwashing (T3) | 0.000 | <0.001 | >-0.001 | <0.001 | .671 |
| Strictness of Policies (T0)🡪Intention (T1)🡪Monitoring (T3)🡪Handwashing (T3) | <0.001 | <0.001 | >-0.001 | <0.001 | .834 |
| Strictness of Policies (T2)🡪Self-efficacy (T3)🡪Handwashing (T3) | **-0.001** | **<0.001** | **-0.001** | **>-0.001** | **<.001** |
| Strictness of Policies (T2)🡪Self-efficacy (T3)🡪Planning (T3)🡪Handwashing (T3) | **>-0.001** | **<0.001** | **>-0.001** | **>-0.001** | **.019** |
| Strictness of Policies (T2)🡪Self-efficacy (T3)🡪Monitoring (T3)🡪Handwashing (T3) | **>-0.001** | **<0.001** | **>-0.001** | **>-0.001** | **<.001** |
| Strictness of Policies (T2)🡪Planning (T3)🡪 Handwashing (T3) | >-0.001 | <0.001 | >-0.001 | <0.001 | .144 |
| Strictness of Policies (T2)🡪Monitoring (T3)🡪 Handwashing (T3) | **-0.001** | **<0.001** | **-0.001** | **>-0.001** | **.012** |

*Note.* The values of the majority of indirect effect estimates were either larger than -0.001 (i.e., -0.0002) or smaller than 0.001 (i.e., 0.0002). Indirect effect estimates presented in bold have values of two-tailed bias corrected confidence intervals that do not include zero. Each bootstrap was based on 5,000 repetitions. 95%CI = two-tailed 95% bias-corrected confidence intervals. CI that do not include zero indicate a significant indirect effect. T0 = Time 0; T1 = Time 1 (1-7 days later); T2= Time 2 (one month after T1), T3= Time 3 (1-7 days after T2); Strictness of Policies = Strictness of Containment and Health Policies; Handwashing = Handwashing Adherence Index (based on the WHO Guidelines).

Data were collected in 14 countries (Australia, Canada, China, France, Gambia, Germany, Israel, Italy, Malaysia, Poland, Portugal, Romania, Singapore from 25.03.2020 to 20.09.2020.

**Supplementary Table 12**

*Sensitivity Analysis: Indirect Effects for the Mediation Model (N = 2,339) with Dropouts Included*

| **Simple indirect effects** | **Estimate** | ***SE*** | **95%BCI** | |  |
| --- | --- | --- | --- | --- | --- |
|  |  |  | **Lower** | **Upper** | ***p*** |
| Strictness of Policies (T0)🡪Risk perception (T1)🡪Handwashing (T3) | >-0.001 | <0.001 | >-0.001 | <0.001 | .087 |
| Strictness of Policies (T0)🡪Risk perception (T1)🡪Intention (T1)🡪Handwashing (T3) | >-0.001 | <0.001 | >-0.001 | <0.001 | .070 |
| Strictness of Policies (T0)🡪Risk perception (T1)🡪Intention (T1)🡪Planning(T3)🡪Handwashing (T3) | >-0.001 | <0.001 | >-0.001 | <0.001 | .053 |
| Strictness of Policies (T0)🡪Risk perception (T1)🡪Intention (T1)🡪Monitoring(T3)🡪Handwashing (T3) | >-0.001 | <0.001 | >-0.001 | <0.001 | .072 |
| Strictness of Policies (T0)🡪Outcome expectancies (T1)🡪Handwashing (T3) | <0.001 | <0.001 | >-0.001 | <0.001 | .410 |
| Strictness of Policies (T0)🡪Outcome expectancies (T1)🡪Intention (T1)🡪Handwashing (T3) | **>-0.001** | **<0.001** | **>-0.001** | **>-0.001** | **.001** |
| Strictness of Policies (T0)🡪Outcome expectancies (T1)🡪Intention (T1)🡪Planning (T3)🡪Handwashing (T3) | **>-0.001** | **<0.001** | **>-0.001** | **>-0.001** | **.016** |
| Strictness of Policies (T0)🡪Outcome expectancies (T1)🡪Intention (T1)🡪Monitoring(T3)🡪Handwashing (T3) | **>-0.001** | **<0.001** | **>-0.001** | **>-0.001** | **.001** |
| Strictness of Policies (T0)🡪Self-efficacy (T1)🡪Handwashing (T3) | >-0.001 | <0.001 | >-0.001 | <0.001 | .747 |
| Strictness of Policies (T0)🡪Self-efficacy (T1)🡪Intention (T1)🡪Handwashing (T3) | **>-0.001** | **<0.001** | **>-0.001** | **>-0.001** | **.000** |
| Strictness of Policies (T0)🡪Self-efficacy (T1)🡪Intention (T1)🡪Planning (T3)🡪Handwashing (T3) | **>-0.001** | **<0.001** | **>-0.001** | **>-0.001** | **.021** |
| Strictness of Policies (T0)🡪Self-efficacy (T1)🡪Intention (T1)🡪Monitoring (T3)🡪Handwashing (T3) | **>-0.001** | **<0.001** | **>-0.001** | **>-0.001** | **.000** |
| Strictness of Policies (T0)🡪Self-efficacy (T1)🡪Self-efficacy(T3)🡪Handwashing (T3) | **>-0.001** | **<0.001** | **-0.001** | **>-0.001** | **.000** |
| Strictness of Policies (T0)🡪Self-efficacy (T1)🡪Self-efficacy(T3)🡪Planning (T3)🡪Handwashing (T3) | **>-0.001** | **<0.001** | **>-0.001** | **>-0.001** | **.025** |
| Strictness of Policies (T0)🡪Self-efficacy (T1)🡪Self-efficacy(T3)🡪Monitoring (T3)🡪Handwashing (T3) | **>-0.001** | **<0.001** | **>-0.001** | **>-0.001** | **.000** |
| Strictness of Policies (T0)🡪Intention (T1)🡪Handwashing (T3) | >-0.001 | <0.001 | >-0.001 | <0.001 | .392 |
| Strictness of Policies (T0)🡪Intention (T1)🡪Planning (T3)🡪Handwashing (T3) | >-0.001 | <0.001 | >-0.001 | <0.001 | .288 |
| Strictness of Policies (T0)🡪Intention (T1)🡪Monitoring (T3)🡪Handwashing (T3) | >-0.001 | <0.001 | >-0.001 | <0.001 | .405 |
| Strictness of Policies (T2)🡪Self-efficacy (T3)🡪Handwashing (T3) | **-0.001** | **<0.001** | **-0.001** | **>-0.001** | **.000** |
| Strictness of Policies (T2)🡪Self-efficacy (T3)🡪Planning (T3)🡪Handwashing (T3) | **>-0.001** | **<0.001** | **>-0.001** | **>-0.001** | **.022** |
| Strictness of Policies (T2)🡪Self-efficacy (T3)🡪Monitoring (T3)🡪Handwashing (T3) | **>-0.001** | **<0.001** | **>-0.001** | **>-0.001** | **.000** |
| Strictness of Policies (T2)🡪Planning (T3)🡪 Handwashing (T3) | >-0.001 | <0.001 | >-0.001 | <0.001 | .122 |
| Strictness of Policies (T2)🡪Monitoring (T3)🡪 Handwashing (T3) | **-0.001** | **<0.001** | **-0.001** | **>-0.001** | **.010** |

*Note.* The values of the majority of indirect effect estimates were either larger than -0.001 (i.e., -0.0002) or smaller than 0.001 (i.e., 0.0002). Indirect effect estimates presented in bold have values of two-tailed bias corrected confidence intervals that do not include zero. Each bootstrap was based on 5,000 repetitions. 95% CI = two-tailed bias-corrected confidence intervals. CI that do not include zero indicate a significant indirect effect. T0 = Time 0; T1 = Time 1 (1-7 days later); T2= Time 2 (one month after T1), T3= Time 3 (1-7 days after T2); Strictness of Policies = Strictness of Containment and Health Policies; Handwashing = Handwashing Adherence Index (based on the WHO Guidelines).

Data were collected in 14 countries (Australia, Canada, China, France, Gambia, Germany, Israel, Italy, Malaysia, Poland, Portugal, Romania, Singapore, and Switzerland) between (25.03.2020-20.09.2020) during the COVID-19 pandemic.

**Supplementary Table 13**

*Sensitivity Analysis: Direct Effects for the Mediation Model (N = 2,339) with Dropouts Included*

| **Variables and hypothesized associations** | **Beta** | **95% lower CI for beta** | **95% upper CI for beta** | ***P*** |
| --- | --- | --- | --- | --- |
| Strictness of Policies (T0) 🡪 Risk Perception (T1) | -.031 | -.069 | .009 | .132 |
| Strictness of Policies (T0) 🡪 Outcome expectancies (T1) | **-.063** | **-.103** | **-.022** | **.002** |
| Strictness of Policies (T0) 🡪 Self-efficacy (T1) | **-.109** | **-.147** | **-.070** | **< .001** |
| Strictness of Policies (T0) 🡪 Intention (T1) | -.013 | -.046 | .019 | .453 |
| Strictness of Policies (T0) 🡪 Planning (T1) | -.036 | -.077 | .007 | .077 |
| Strictness of Policies (T0) 🡪 Monitoring (T1) | **-.095** | **-.135** | **-.052** | **< .001** |
| Strictness of Policies (T0) 🡪 Handwashing (T1) | -.021 | -.058 | .018 | .251 |
| Strictness of Policies (T0) 🡪 Strictness of Policies (T2) | **.414** | **.371** | **.452** | **< .001** |
| Risk Perception (T1) 🡪 Intention (T1) | **.069** | **.036** | **.102** | **< .001** |
| Risk Perception (T1) 🡪 Handwashing (T3) | .022 | -.002 | .045 | .073 |
| Outcome expectancies (T1) 🡪 Intention (T1) | **.250** | **.211** | **.288** | **< .001** |
| Outcome expectancies (T1) 🡪 Handwashing (T3) | -.010 | -.038 | .018 | .497 |
| Self-efficacy (T1) 🡪 Intention (T1) | **.405** | **.363** | **.446** | **< .001** |
| Self-efficacy (T1) 🡪 Self-efficacy (T3) | **.659** | **.621** | **.694** | **< .001** |
| Self-efficacy (T1) 🡪 Handwashing (T3) | .007 | -.040 | .052 | .702 |
| Intention (T1) 🡪 Handwashing (T1) | **.746** | **.673** | **.820** | **< .001** |
| Intention (T1) 🡪 Planning (T3) | **.090** | **.057** | **.123** | **< .001** |
| Intention (T1) 🡪 Monitoring (T3) | **.159** | **.122** | **.198** | **< .001** |
| Intention (T1) 🡪 Handwashing (T3) | **.068** | **.036** | **.099** | **< .001** |
| Planning (T1) 🡪 Planning (T3) | **.717** | **.689** | **.741** | **< .001** |
| Planning (T1) 🡪 Handwashing (T3) | -.041 | -.092 | .005 | .084 |
| Monitoring (T1) 🡪 Monitoring (T3) | **.553** | **.514** | **.589** | **< .001** |
| Monitoring (T1) 🡪 Handwashing (T3) | -.042 | -.085 | .004 | .074 |
| Handwashing (T1) 🡪 Handwashing (T3) | **.590** | **.550** | **.627** | **< .001** |
| Strictness of Policies (T2) 🡪 Self-efficacy (T3) | **-.089** | **-.126** | **-.050** | **< .001** |
| Strictness of Policies (T2) 🡪 Planning (T3) | -.021 | -.052 | .012 | .088 |
| Strictness of Policies (T2) 🡪 Monitoring (T3) | **-.045** | **-.080** | **-.011** | **< .001** |
| Strictness of Policies (T2) 🡪 Handwashing (T3) | **-.033** | **-.064** | **-.002** | **.005** |
| Self-efficacy (T3) 🡪 Planning (T3) | **.143** | **.107** | **.178** | **< .001** |
| Self-efficacy (T3) 🡪 Monitoring (T3) | **.218** | **.173** | **.262** | **< .001** |
| Self-efficacy (T3) 🡪 Handwashing (T3) | **.145** | **.091** | **.201** | **< .001** |
| Planning (T3) 🡪 Handwashing (T3) | **.059** | **.004** | **.114** | **.005** |
| Monitoring (T3) 🡪 Handwashing (T3) | **.220** | **.162** | **.274** | **< .001** |

*Note.* Direct effect estimates presented in bold have values of two-tailed bias corrected confidence intervals that do not include zero. T0 = Time 0; T1 = Time 1 (1-7 days later); T2= Time 2 (one month after T1), T3= Time 3 (1-7 days after T2); Strictness of Policies = Strictness of Containment and Health Policies; Handwashing = Handwashing Adherence Index (based on the WHO Guidelines). Data were collected in 14 countries (Australia, Canada, China, France, Gambia, Germany, Israel, Italy, Malaysia, Poland, Portugal, Romania, Singapore, and Switzerland) from 25.03.2020 to 20.09.2020.

**Supplementary Table 14**

*Sensitivity Analysis: Covariances for the Mediation Model (N = 2,339) with Dropouts Included*

| **Covariances** | Estimate | *SE* | *p* |
| --- | --- | --- | --- |
| Outcome expectancies (T1) 🡨🡪 Risk Perception (T1) | -0.003 | 0.009 | .764 |
| Self-efficacy (T1) 🡨🡪 Risk Perception (T1) | 0.050 | 0.010 | < .001 |
| Intention (T1) 🡨🡪 Planning (T1) | 0.103 | 0.007 | < .001 |
| Intention (T1) 🡨🡪 Monitoring (T1) | 0.094 | 0.006 | < .001 |
| Planning (T1) 🡨🡪 Risk Perception (T1) | 0.013 | 0.012 | .273 |
| Planning (T1) 🡨🡪 Outcome expectancies (T1) | 0.146 | 0.008 | < .001 |
| Planning (T1) 🡨🡪 Self-efficacy (T1) | 0.125 | 0.009 | < .001 |
| Monitoring (T1) 🡨🡪 Risk Perception (T1) | 0.044 | 0.010 | < .001 |
| Monitoring (T1) 🡨🡪 Outcome expectancies (T1) | 0.120 | 0.007 | < .001 |
| Monitoring (T1) 🡨🡪 Self-efficacy (T1) | 0.157 | 0.008 | < .001 |
| Monitoring (T1) 🡨🡪 Planning (T3) | 0.185 | 0.009 | < .001 |
| Handwashing (T1) 🡨🡪 Risk Perception (T1) | 0.003 | 0.009 | .758 |
| Handwashing (T1) 🡨🡪 Outcome expectancies (T1) | 0.007 | 0.006 | .231 |
| Handwashing (T1) 🡨🡪 Self-efficacy (T1) | 0.019 | 0.008 | .014 |
| Handwashing (T1) 🡨🡪 Monitoring (T1) | 0.041 | 0.007 | < .001 |
| Handwashing (T1) 🡨🡪 Intention (T1) | -0.100 | 0.011 | < .001 |
| Planning (T3) 🡨🡪 Monitoring (T3) | 0.024 | 0.002 | < .001 |

*Note.* T0 = Time 0; T1 = Time 1 (1-7 days later); T2= Time 2 (one month after T1), T3= Time 3 (1-7 days after T2); Strictness of Policies = Strictness of Containment and Health Policies; Handwashing = Handwashing Adherence Index (based on the WHO Guidelines). Data were collected in 14 countries (Australia, Canada, China, France, Gambia, Germany, Israel, Italy, Malaysia, Poland, Portugal, Romania, Singapore, and Switzerland) from 25.03.2020 to 20.09.2020.

**Supplementary Table 15**

*Correlation Coefficients for Associations Between the Variables Included in the Hypothetical Model, Calculated for Each Country Separately*

| **Variables and hypothesized associations** | **Canada** | **China** | **France** | **Germany** | **Israel** | **Italy** | **Malaysia** |
| --- | --- | --- | --- | --- | --- | --- | --- |
| Strictness of Policies (T0) 🡨🡪 Strictness of Policies (T2) | **.680** | **-.373** | **.403** | **-.193** | **.583** | **.412** | **-.387** |
| Strictness of Policies (T0) 🡨🡪 Risk Perception (T1) | .080 | .105 | .103 | .078 | .062 | -.150 | -.259 |
| Strictness of Policies (T0)🡨 🡪 Outcome expectancies (T1) | -.142 | .081 | -.025 | -.050 | -.014 | .163 | .049 |
| Strictness of Policies (T0) 🡨🡪 Self-efficacy (T1) | -.018 | -.064 | .022 | .019 | -.046 | .135 | -.119 |
| Strictness of Policies (T0) 🡨🡪 Intention (T1) | .105 | -.161 | -.071 | -.017 | .088 | .008 | .084 |
| Strictness of Policies (T0) 🡨🡪 Planning (T1) | .051 | -.074 | .041 | -.096 | -.113 | .179 | .081 |
| Strictness of Policies (T0) 🡨🡪 Monitoring (T1) | .117 | -.082 | .090 | .060 | .075 | .123 | .081 |
| Strictness of Policies (T0) 🡨🡪 Handwashing (T1) | -.011 | -.111 | -.080 | -.036 | .163 | -.131 | .148 |
| Risk Perception (T1) 🡨🡪 Intention (T1) | .066 | -.168 | .107 | -.002 | .134 | .208 | .222 |
| Risk Perception (T1) 🡨🡪 Handwashing (T3) | .240 | -.088 | .096 | .079 | .086 | .004 | .279 |
| Outcome expectancies (T1) 🡨🡪 Intention (T1) | **.401** | **.259** | **.211** | **.548** | **.364** | **.366** | **.506** |
| Outcome expectancies (T1)🡨 🡪 Handwashing (T3) | **.369** | **.260** | **.214** | **.259** | **.222** | **.322** | .212 |
| Self-efficacy (T1) 🡨🡪 Intention (T1) | **.555** | **.534** | **.479** | **.441** | **.537** | **.618** | **.599** |
| Self-efficacy (T1) 🡨🡪 Self-efficacy (T3) | .164 | **.602** | **.551** | **.494** | **.582** | **.591** | **.463** |
| Self-efficacy (T1) 🡨🡪 Handwashing (T3) | **.340** | **.411** | **.451** | **.340** | **.466** | **.460** | **.348** |
| Intention (T1) 🡨🡪 Handwashing (T1) | **.682** | **.463** | **.671** | **.413** | **.610** | **.462** | **.476** |
| Intention (T1) 🡨🡪 Planning (T3) | **.498** | **.383** | **.389** | **.449** | **.275** | **.465** | **.357** |
| Intention (T1) 🡨🡪 Monitoring (T3) | **.561** | **.307** | **.453** | **.525** | **.444** | **.492** | **.349** |
| Intention (T1) 🡨🡪 Handwashing (T3) | **.570** | **.375** | **.600** | **.403** | **.565** | **.415** | .307 |
| Planning (T1) 🡨🡪 Planning (T3) | **.760** | **.648** | **.678** | **.668** | **.464** | **.700** | **.545** |
| Planning (T1) 🡨🡪 Handwashing (T3) | **.649** | **.365** | **.360** | **.363** | .100 | **.253** | .167 |
| Monitoring (T1) 🡨🡪 Monitoring (T3) | **.615** | **.423** | **.723** | **.671** | **.563** | **.640** | **.385** |
| Monitoring (T1) 🡨🡪 Handwashing (T3) | **.604** | **.410** | **.546** | **.532** | **.437** | **.297** | .089 |
| Handwashing (T1) 🡨🡪 Handwashing (T3) | **.778** | **.472** | **.790** | **.588** | **.638** | **.684** | **.491** |
| Strictness of Policies (T2) 🡨🡪 Self-efficacy (T3) | -.069 | -.009 | .019 | .114 | .030 | -.004 | -.074 |
| Strictness of Policies (T2) 🡨🡪 Planning (T3) | .045 | -.037 | -.044 | **.177** | -.176 | -.089 | -.175 |
| Strictness of Policies (T2) 🡨🡪 Monitoring (T3) | .106 | -.106 | .030 | .065 | -.011 | .078 | .158 |
| Strictness of Policies (T2) 🡨🡪 Handwashing (T3) | -.023 | .012 | .004 | .134 | **.252** | .068 | .024 |
| Self-efficacy (T3) 🡨🡪 Planning (T3) | .026 | **.437** | **.344** | **.498** | **.382** | **.479** | **.561** |
| Self-efficacy (T3) 🡨🡪 Monitoring (T3) | .081 | **.464** | **.532** | **.566** | **.458** | **.594** | **.554** |
| Self-efficacy (T3) 🡨🡪 Handwashing (T3) | .180 | **.432** | **.560** | **.481** | **.556** | **.581** | **.647** |
| Planning (T3) 🡨🡪 Handwashing (T3) | **.475** | **.455** | **.409** | **.439** | **.212** | **.315** | **.376** |
| Monitoring (T3) 🡨🡪 Handwashing (T3) | **.704** | **.515** | **.606** | **.503** | **.547** | **.502** | **.391** |

*Note.* Significant coefficients at *p* < .05 are marked in bold. T0 = Time 0; T1 = Time 1 (1-7 days later); T2= Time 2 (one month after T1), T3= Time 3 (1-7 days after T2); Strictness of Policies = Strictness of Containment and Health Policies; Handwashing = Handwashing Adherence Index (based on the WHO Guidelines); Data were collected in 14 countries (Australia, Canada, China, France, Gambia, Germany, Israel, Italy, Malaysia, Poland, Portugal, Romania, Singapore, and Switzerland) from 25.03.2020 to 20.09.2020.

**Supplementary Table 15 – cont.**

*Correlation Coefficients for Associations Between the Variables Included in the Hypothetical Model, Calculated for Each Country Separately*

| **Variables and hypothesized associations** | **Poland** | **Portugal** | **Singapore** | **Switzerland** | **Australia** | **Romania** | **Gambia** |
| --- | --- | --- | --- | --- | --- | --- | --- |
| Strictness of Policies (T0) 🡨🡪 Strictness of Policies (T2) | **-.630** | -.072 | **.703** | **.287** | **-.149** | **.466** | -.208 |
| Strictness of Policies (T0) 🡨🡪 Risk Perception (T1) | .021 | .167 | .075 | .066 | -.125 | -.193 | .140 |
| Strictness of Policies (T0)🡨 🡪 Outcome expectancies (T1) | -.129 | .048 | -.070 | **.186** | .091 | .158 | .177 |
| Strictness of Policies (T0) 🡨🡪 Self-efficacy (T1) | -.089 | .026 | -.042 | .090 | .126 | .180 | .195 |
| Strictness of Policies (T0) 🡨🡪 Intention (T1) | -.103 | .092 | -.478 | .025 | .036 | .098 | -.218 |
| Strictness of Policies (T0) 🡨🡪 Planning (T1) | -.216 | -.199 | -.106 | -.079 | .031 | -.037 | -.394 |
| Strictness of Policies (T0) 🡨🡪 Monitoring (T1) | **-.283** | -.234 | -.367 | -.036 | -.015 | -.070 | -.050 |
| Strictness of Policies (T0) 🡨🡪 Handwashing (T1) | .026 | -.025 | -.371 | -.014 | .033 | -.081 | .189 |
| Risk Perception (T1) 🡨🡪 Intention (T1) | .127 | .147 | .188 | .145 | .012 | -.038 | -.025 |
| Risk Perception (T1) 🡨🡪 Handwashing (T3) | .061 | .101 | .329 | .155 | .114 | .058 | .202 |
| Outcome expectancies (T1) 🡨🡪 Intention (T1) | **.454** | **.440** | **.769** | **.435** | **.460** | **.387** | **.416** |
| Outcome expectancies (T1)🡨 🡪 Handwashing (T3) | .223 | .218 | **.640** | **.425** | .136 | .080 | -.031 |
| Self-efficacy (T1) 🡨🡪 Intention (T1) | **.652** | **.578** | .086 | **.434** | **.484** | **.370** | **.613** |
| Self-efficacy (T1) 🡨🡪 Self-efficacy (T3) | **.476** | **.535** | .240 | **.591** | **.385** | **.361** | **.767** |
| Self-efficacy (T1) 🡨🡪 Handwashing (T3) | **.541** | **.386** | -.057 | **.387** | **.261** | **.288** | .079 |
| Intention (T1) 🡨🡪 Handwashing (T1) | **.495** | **.456** | **.800** | **.481** | **.631** | **.240** | **.**288 |
| Intention (T1) 🡨🡪 Monitoring (T3) | **.617** | **.397** | **.703** | **.456** | **.524** | **.252** | .360 |
| Intention (T1) 🡨🡪 Planning (T3) | **.508** | .178 | .364 | **.483** | **.358** | **.302** | .175 |
| Intention (T1) 🡨🡪 Handwashing (T3) | **.471** | **.467** | **.728** | **.497** | **.492** | .079 | .097 |
| Planning (T1) 🡨🡪 Planning (T3) | **.782** | **.647** | .360 | **.764** | **.609** | **.710** | **.422** |
| Planning (T1) 🡨🡪 Handwashing (T3) | **.301** | .199 | **.700** | **.299** | **.375** | **.245** | -.026 |
| Monitoring (T1) 🡨🡪 Monitoring (T3) | **.697** | .177 | **.674** | **.692** | **.632** | **.543** | .372 |
| Monitoring (T1) 🡨🡪 Handwashing (T3) | **.413** | .209 | .296 | **.467** | **.409** | **.246** | .318 |
| Handwashing (T1) 🡨🡪 Handwashing (T3) | **.679** | **.672** | **.808** | **.724** | **.717** | **.633** | **.**296 |
| Strictness of Policies (T2) 🡨🡪 Self-efficacy (T3) | -.004 | .057 | **.595** | .041 | .115 | .131 | .333 |
| Strictness of Policies (T2) 🡨🡪 Planning (T3) | .153 | -.104 | -.100 | -.073 | .043 | -.062 | .246 |
| Strictness of Policies (T2) 🡨🡪 Monitoring (T3) | .151 | -.179 | -.512 | -.099 | .007 | -.138 | .075 |
| Strictness of Policies (T2) 🡨🡪 Handwashing (T3) | -.083 | .113 | -.065 | -.060 | .019 | -.113 | -.228 |
| Self-efficacy (T3) 🡨🡪 Planning (T3) | **.250** | .232 | .440 | **.399** | **.284** | **.403** | .153 |
| Self-efficacy (T3) 🡨🡪 Monitoring (T3) | **.493** | **.426** | -.055 | **.454** | **.398** | **.404** | .302 |
| Self-efficacy (T3) 🡨🡪 Handwashing (T3) | **.247** | **.577** | .523 | **.547** | **.378** | **.246** | .171 |
| Planning (T3) 🡨🡪 Handwashing (T3) | **.364** | **.313** | .184 | **.428** | **.457** | .123 | .224 |
| Monitoring (T3) 🡨🡪 Handwashing (T3) | **.492** | **.594** | .514 | **.587** | **.601** | .210 | **.504** |

*Note.* Significant coefficients at *p* < .05 are marked in bold. T0 = Time 0; T1 = Time 1 (1-7 days later); T2= Time 2 (one month after T1), T3= Time 3 (1-7 days after T2); Strictness of Policies = Strictness of Containment and Health Policies; Handwashing = Handwashing Adherence Index (based on the WHO Guidelines); Data were collected in 14 countries (Australia, Canada, China, France, Gambia, Germany, Israel, Italy, Malaysia, Poland, Portugal, Romania, Singapore, and Switzerland) from 25.03.2020 to 20.09.2020.
